# Spirit of the Foreign Periodicals, &c.

**Published:** 1839-04-01

**Authors:** 


### Spirit of the Foreign Periodicals, &c.

### On Vaccination and Small-pox.

In the last number of this Journal (vide page 271) we commenced an article on the present state of Vaccination in different countries of Europe, and, as it was left unfinished, we promised to continue the subject at our next trimestral appearance.

We stated the prevailing doctrines and practice of the German physicians, more especially in reference to the question of Re-vaccination, which has been adopted so extensively in the armies of Prussia and of some other states. We now proceed to explain what has been done and observed elsewhere; and shall then close our remarks with a series of corollaries, embodying most of the important information which has hitherto been obtained.

In no country has vaccination been prosecuted with more enlightened and assiduous zeal than in Denmark. The reports from this, as well as from every other country where it has been introduced, concur in loudly proclaiming the benefits of the great discovery; and, as very exact tables of mortality seem to be kept in Copenhagen, we have been enabled to ascertain the following particulars, in reference to the question now under consideration.

From the year 1800 to 1804, not a single case of small-pox in a vaccinated person was observed in that city. In 1804, two cases only occurred; and in both of these the disease exhibited a modified or *varioloid* character. In 1805, five persons (vaccinated, we presume) died from small-pox. In 1806, three vaccinated persons died. In 1808, there were 46 deaths in all from small-pox; and, of this number, 13 occurred in persons who were reputed to have been regularly vaccinated. In 1819, the number of cases of small pox among vaccinated as well as among unvaccinated persons had very considerably increased; and in 1823, there was a regular epidemic of the disease in most parts of the kingdom.

During the three subsequent years—from 1824 to 1827—there was an annual renewal of the epidemic; and the following particulars are derived from the reports of Dr. Mæhl, physician of the small-pox hospital at Copenhagen. From January 1824 to February 1825, there were admitted 412 cases of genuine and modified small-pox. Of these 412 cases, 257 occurred in persons who had been vaccinated, 58 in persons who had passed through small-pox before, and 97 in persons who had not had either the cow-pox or the small-pox.

The ages of the 257 patients it is important to attend to, as we are thus enabled to form some idea of the length of time that the protecting influence of vaccination may be supposed to last:—

| | |
|---|---|
| 24 | were under seven years of age. |
| 42 | were between seven and eleven years of age. |
| 191 | were between twelve and twenty-three years of age. |
| 257 | |

So much for the age of the patients; and now for the character and issue of the disease. Of the whole 257 cases, there were only 16 of genuine small-pox; in all the rest the disease was more or less decidedly modified. Of the 16 cases, only 3 proved fatal; and these three occurred among adults.

It is needless to point out to the reader's attention how these facts strongly bear out the idea, now generally received, that the protecting influence of vaccination decreases progressively with the length of the time that has elapsed since it was performed. The number of deaths among the remaining 155 cases was 37. (It is not stated whether any of these cases occurred among the 58 patients, who had been previously affected with the small-pox.)

In the following epidemic, which commenced in September 1825, and did not cease till the middle of 1827, 625 cases of *variolous* and *varioloid* disease were admitted. Of these, there occurred in persons who had been vaccinated 438 cases,* in 26 of which the disease was genuine variola; two of these proved fatal. By far the greater number of the 438 patients were upwards of ten years of age.

The next epidemic made its appearance in March 1828 and continued, with only a few short interruptions, to July 1830. During this period, 562 patients affected with *variolous* or *varioloid* disease were admitted. Of these, there were 111 cases of genuine variola, and 28 of them proved fatal. In 29 of the 111 cases, the patients had been vaccinated. The deaths were 24 among the unvaccinated, and 4 among the vaccinated patients—the ratio being about 1 in 3 among the former, and 1 in 7½ among the latter.

Of the 29 vaccinated patients, all, with the exception of one only, were adults —thus affording another proof that the protective influence of the cow-pox ceases only after the lapse of a considerable number of years.

In August 1832, commenced the most violent epidemic of small-pox in Copenhagen, which had been known since the introduction of vaccination. From that period till towards the close of 1834, 1045 patients were received into the hospital. Of this number, 898 had been vaccinated, and 147 had not. Of the former, 10 only died; whereas among the latter, there were 34 deaths.

Of the 1045 cases, there were 179 of genuine small-pox, of which 119 occurred in unvaccinated and 60 in vaccinated persons; the remaining number exhibiting the varioloid character.

Not one of the cases of the genuine disease occurred in a vaccinated patient under 14 years of age; and every one of the 10 patients who died was above 22 years of age.

To these facts it may be added that not a single instance of variolous or of varioloid disease during this and the preceding epidemic was observed among any who had been re-vaccinated.†

During the year 1835, small-pox continued to prevail very extensively in Copenhagen. From May till the end of December 1197 cases were admitted. Of these, 1043 occurred in vaccinated, and 125 in unvaccinated persons; and as to the remaining 31 patients, it could not be accurately ascertained whether they had had either the cow-pox or small-pox before.

Although, in a great majority of the vaccinated patients, the disease was mild and of the varioloid type, the cases were not rare in which all the characters of genuine small-pox, and even of the confluent kind, were observed. There were 47 deaths among the 1043 vaccinated patients; and all of them occurred in persons between 19 and 35 years of age.

Of the 123 cases which occurred in unvaccinated persons, (some of whom had had small-pox in their infancy) 51 proved fatal. Among these, five occurred in infants, two in children below 10 years of age, and the rest in adults.

---

* We were somewhat surprised at first on finding such a large proportion of the cases occurring in vaccinated persons. But this is probably attributable to the universal adoption of vaccination among all classes in Denmark, and to its consequent failure in numerous instances. If we are not mistaken, it is rendered imperative by the Government; but the operation may be performed by clergymen, midwives, and many other unprofessional persons.

† The Danish Government has, within the last few years, ordered that all the soldiers of their army and all fresh recruits be re-vaccinated.

Again it was observed that all persons, who had been re-vaccinated, escaped the epidemic contagion.

We shall now briefly direct our attention to the state of one or two other countries in Europe, in reference to the march of variolous disease, and the influence which vaccination has exerted upon it. It is most gratifying to observe the uniform and invariable decrease in the ravages of small-pox after the first introduction of vaccination, throughout every country without exception; and to find that the medical men in all parts of the globe bear the same unequivocal testimony in its favour.

In Sweden the decrease of the mortality from small-pox may be judged of by the following table:

From 1782 to 1791, 47,587 deaths.
From 1792 to 1801, 44,184 —
From 1802 to 1811, 14,904 —
From 1812 to 1821, 3,309 —

In 1822, the mortality seems to have fallen to its *minimum*. After this year, the small-pox began to regain, in part, its lost ground. In 1824, there were 560 fatal cases; of these, 103 occurred in persons who were supposed to have been vaccinated.

Every one of these 103 patients was upwards of 15 years of age; whereas among the other 457 fatal cases (in unvaccinated individuals) 229 occurred in children under two years of age, 162 in those between 2 and 15 years, 98 in persons between 15 and 25 years, and the remaining 71 in persons above the latter age.*

Here M. *Dezeimeris* (from whose paper, in a recent number of the French Journal *L'Experience*, we have derived most of the preceding data) remarks that, after examining these and similar documents, it is impossible to escape from the conclusion that vaccination is *absolutely preservative* against small-pox contagion for about ten years after it has been performed, *simply protective* for a few years longer, and at length that it is *insufficient* to prevent even a fatal attack of the disease. He adds that, during this epidemic, the number of second attacks of small-pox—most of which were very severe—was not much inferior to the number of fatal cases occurring in vaccinated persons.

In the years 1831 and 1832 there was another wide-spread epidemic of small-pox in Sweden. Now mark the conclusion which Dr. *Westman*, the physician of the Provisional Small-pox Hospital at Stockholm, deduced from his extended observations:

*The disease was violent in adults, who had been vaccinated in their infancy; it exhibited more and more distinctly the modified character and was milder and milder in proportion as the patients had been more and more recently vaccinated; and lastly children, who had been vaccinated a short time before the invasion of the epidemic, altogether escaped.*

So much for what has been observed in Sweden. Let us now hear what has been the state of vaccination and of small-pox epidemics in the adjacent country of Norway. Although vaccination was practised there since the close of the last century, it was not till 1810 that it was made imperative upon all by an edict of the government. From this period to about 1819, no variolous epidemic was known in the country. But ever since the latter year, the genuine small-pox and also the various forms of varioloid disease have been progressively on the increase.

The prevailing opinion among the Norwegian physicians at present is, that genuine small-pox seldom or never attacks those persons, in whom vaccination

* The reader will probably remark an incongruity between the sum total of the separate numbers and the aggregate number of 560 mentioned at first.—*Rev.*

had produced a normal and perfect eruption of vesicles on the arm; but that such persons may however contract a varioloid disease.

Dr. Holtz, one of their ablest physicians and a professor in the University of Christiania, in adopting these views, has very properly alluded to the extreme difficulty of ascertaining exactly what had been the actual condition of the vaccine vesicles in many persons, who have subsequently suffered from the small-pox contagion.

A great number, especially in the country, have received certificates of having been vaccinated, although they had never been examined by the medical man after the operation was performed.

The Norwegian physicians are inclined to the opinion that the vaccine virus has lost a certain degree of its active powers in passing successively from one individual to another; and they therefore recommend that a fresh supply should be occasionally taken from the cow itself. It would seem that the disease has never been found upon that animal in Norway; and hence the virus in use there has always been derived from other countries.

We have already stated that vaccination is made obligatory upon all in Norway. No one can be admitted into any public establishment, nor even confirmed nor married, without producing a certificate of having been vaccinated. Moreover, the inoculation of small-pox is prohibited; and any person practising it is liable to severe punishment.

Re-vaccination has not yet been *ordonnée* in Sweden and Norway by a public edict; but it has been pretty extensively practised by some of the medical men in both countries; and it is generally believed that, if it was universally adopted, not only the *variolous* but also the *varioloid* contagion might be arrested.

In France, very little comparatively has been done to advance our knowledge on the various important topics connected with vaccination. The general question has however very recently come under the consideration of the Royal Academy of Medicine, in consequence of its having been made, last year, the *theme* for one of the great *Monthyon* prizes, which are annually awarded to the best essay on some medical subject.

It would seem that re-vaccination has been hitherto very little practised in France; but it is right to state that some of the most experienced and practical members of the Academy, such as M. *Bousquet*, the head of the vaccine board, and MM. Chomel, Andral, and Bouillaud, all concurred in recommending the practice, at a recent *séance* of that learned body.

There was considerable difference of opinion expressed as to whether the vaccine lymph has become deteriorated of late years; and particular stress was laid on the results of the experiments, which were a short time ago performed by M. *Bousquet* with virus obtained directly from the cow.*

Lastly, we must briefly allude to what has been recently observed at home on the important questions connected with vaccination.

From the last report by Dr. Gregory of the Small-pox Hospital in London, it appears that, during the year 1838, 694 cases of small-pox were admitted; besides which, more than 100 persons affected with the disease were refused admission from want of accommodation.

Of the 694 cases, 298 occurred in persons reputed to have been vaccinated; and of these 298, 114 exhibited the mild and safe form of the disease called the varicelloid: in 66 the disease was severe at first, but shortened and modified in its subsequent stages; and in the remaining 118 (or 40 per cent.) it presented the regular, normal, or unmitigated form; and in these last the rate of mortality was nearly the same as in patients who had never been vaccinated.

"The deaths in this class amounted to 31; but from them some deductions must

---

* Vide Medico-Chirurgical Review, April, 1837.

in fairness be made, for, in the Spring of the year, from the crowded state of the wards, fever of a very malignant character pervaded the hospital, affecting indiscriminately those who had, and those who had not, been vaccinated. To that cause, and to superadded erysipelas, the deaths of 8 or 10 among the vaccinated may be attributed, leaving the total mortality by small-pox after vaccination, 21 out of 298, or 7 per cent.

The following table, shewing the ages of the patients admitted during the last year, distinguishing the vaccinated from the unvaccinated, affords some interesting results :

*Table, shewing the Ages of the Patients admitted into the Small-pox Hospital in 1838, with the Mortality among the same, distinguishing the vaccinated from the unvaccinated.*

| Ages. | Unvaccinated. | | Vaccinated. | |
|---|---|---|---|---|
| | Admitted. | Died. | Admitted. | Died. |
| Under 5 years of age . . | 42 | 20 | 0 | 0 |
| From 5 to 9 inclusive | 37 | 11 | 5 | 0 |
| „ 10 to 14 „ | 30 | 8 | 25 | 0 |
| „ 15 to 19 „ | 104 | 32 | 90 | 6 |
| „ 20 to 24 „ | 115 | 50 | 106 | 16 |
| „ 25 to 30 „ | 45 | 23 | 55 | 8 |
| „ 31 to 35 „ | 12 | 7 | 13 | 1 |
| Above 35 years of Age. | 11 | 6 | 4 | 0 |
| Total | 396 | 157 | 298 | 31* |

From this table it appears, that in the early periods of life the preventive powers of vaccination are almost complete; 5 vaccinated children only, under the age of 10, having been received; while of the unvaccinated, within the same ages, the number admitted was 79, of whom 31 died. At 10 years of age the receptivity of the small-pox in the vaccinated may be said to commence, but the disorder then shews itself only in its mildest form. From 15 to 25 years of age the disposition of the body to receive small-pox after vaccination increases, and the severity of the disease augments in a like ratio. The table indicates a decline in the number attacked by small-pox after the age of 25, but the diminution is equally great among the unprotected as among the vaccinated, and may, therefore, be owing to causes independent of vaccine agency."

With respect to the propriety of general Re-vaccination, Dr. Gregory is quite inclined to approve of the practice, and recommends that it should be done at from 15 to 20 years after the date of the first operation.

Having thus, briefly indeed, stated some of the most important facts, observed of recent years in different countries of Europe, respecting vaccination and small-pox, we now submit to our readers' notice the following propositions or corollaries, as affording in some degree a *precis* or abstract of the most

* Of these 6 died of typhus, 3 of superadded erysipelas, 1 of chronic diarrhœa. Total 10.

valuable conclusions on the subject, which may be drawn from the writers of the best established reputation.

1. There is a close affinity between the nature of small-pox and that of cow-pox. Dr. Jenner regarded the one as a mere variety or modification of the other; both having, according to him, the same origin, and being subject to the same laws. Hence he always called the latter *variolæ vaccinæ.**

2. As it is well known that small-pox may affect the same individual twice, it is only what we might expect that cow-pox, which is the milder form of the disease, is not an infallible preservative against variolous contagion.†

3. The virus both of small-pox and of cow-pox seems to undergo certain changes in the systems of some persons, becoming either more or less powerfully energetic than usual; and the virus, taken from such persons, may be used to propagate to other persons a more or less active form of either disease.

The late Dr. Adams succeeded in producing a benign form of variola, attended with scarcely any eruption and little or no constitutional affection, by selecting the virus from such patients as exhibited a mild form of small-pox, which occasionally shewed itself in London; and, on the other hand, it has been found that vaccination produces in certain individuals a more than usually active or intense kind of cow-pox vesicle, the lymph of which may be communicated to and re-produced in others, and which *very probably* insures a more than ordinarily complete protection against variolous contagion.

It thus appears that there are various *grades* of the variolous and of the vaccine virus; some being much more active than others.

4. The developement and maturation of the vaccine vesicle may be much retarded and modified by several causes. The most important of these appear to be dentition, and the existence of herpetic and other forms of cutaneous disease. Under such circumstances, the *pock* is apt to be more or less irregular and imperfect.

It is most important to remark, that "the fluid taken from a spurious or imperfect vaccine vesicle can propagate and perpetuate its like; and, even if it be taken from a genuine vesicle in its far-advanced stages, it is capable of producing varieties, which will become permanent if we continue to employ it."

5. Whenever there is the slightest deviation from the normal or genuine characters of the vaccine vesicle, vaccination should be repeated. The test-operation,

---

* The idea at first entertained by Dr. Jenner, that the disease in the cow is always owing to contagion from horses affected with what is called the *grease*, was soon abandoned by him.

The truth is, that the horse, as well as the cow, is subject to an eruptive disease of a vesicular character during certain variolous epizootics; and the matter of the vesicles on the horse, inoculated upon the human body, will produce as genuine a *cow-pock* as that taken from the teats of the cow.

Dr. Jenner repeatedly used *equine* matter for the purpose of inoculation with complete success. The disease in the horse should be called *variolæ equinæ* and not the *grease*, with which it has no alliance, nor any resemblance, further than that the heels of the animal in both complaints are the seat of ulcerated fissures.

Even poultry seem to suffer occasionally from a variolous epizootic; and it is a curious circumstance that the Hindoos apply the term *gootry* (small-pox) to the disorder. Hence, perhaps, the origin of the word chicken-pox.

† Dr. Baron, in his life of Jenner, mentions a case of extraordinary susceptibility to variolous and vaccine contagion. A child was vaccinated, apparently with success, in India; the operation was repeated on his arrival in England, and again with effect. He was subsequently inoculated for the small-pox, and received the disease; and, after all, he caught the infection in a casual way.

recommended by the late Mr. Bryce of Edinburgh, deserves to be more generally adopted than it has been.

6. The number of punctures to be made on the arm must vary, according as the lymph employed is more or less active. Dr. Heim of Wurtemburg, and, we believe also, Dr. Gregory of London recommend ten or twelve insertions of the lymph in ordinary use. When the lymph is unusually energetic, as seems to be frequently the case when it has been recently derived from the cow, two or three punctures will often produce as much local and constitutional irritation as ten or twelve punctures with the lymph that has been used successively for a number of years. On this subject we must refer to the work of Jenner himself, to the memoir of M. Bousquet (*vide* Med.-Chir. Rev. April 1837), and to the very interesting report of Mr. Macpherson from Moorshedabad (*vide* Trans. of Med. and Phys. Soc. of Calcutta, vol. vi.).

Fresh fluid lymph, taken directly from the vesicles, is always preferable to that which has been kept and become dry; and lymph from a vesicle in its early stage is certainly more active than that from one that is more advanced.

Whatever be the number of punctures made, it is desireable that a certain degree of constitutional disturbance be induced by the operation. It is probable that, when this does not occur, the system is not so thoroughly protected, as when it does.

7. Recurrence should be had occasionally to the cow for fresh supplies of lymph, especially when that, which is in use, appears to lose some degree of its intensity.

It is to be remembered however, that by far the greater number of experiments, made with lymph procured from the cow, have hitherto failed. Whether this arises from the genuine disease in the cow not having been properly discriminated—(for it would seem that the animal is subject to various anomalous eruptive complaints, like to, but in reality different from, the genuine *Variolæ Vaccinæ*)—or from other causes, cannot well be decided. It is a curious fact that in France, upon *one* occasion only since the first introduction of vaccination, has the genuine lymph been derived from its original source: this occurred about three years ago at Passy, near Paris. Dr. Gregory too, in his last Report of the Small-pox Hospital, alludes to the subject in these words: "We have received indeed from Bristol, Aylesbury, and the North of Scotland, supplies of matter recently taken from the Cow, with which experiments have been made, but as none of them appear to exceed, or even to equal in intensity, the Lymph now in use, it has not been thought advisable to make any alteration. Recurrence to the Cow for fresh Lymph is not a measure lightly to be had recourse to, nor should it be advised until the old and tried stock has obviously degenerated."

The question therefore of the propriety of occasionally renewing our supplies of lymph from its original source requires to be more extensively examined, before we can arrive at any positive conclusions on the subject. Still there is information sufficient to warrant us in recommeuding the practice.

8. In taking lymph from the arm of a vaccinated child, one or two of the vesicles should be left unopened and undisturbed.

9. The protective influence of vaccination against small-pox contagion is only temporary or limited to a certain number of years. It appears to remain almost complete for ten or twelve years; and then progressively to become less and less decided.

10. It is therefore proper to have recourse to Re-vaccination. The effects of this operation vary much in different persons. In some, the punctured wounds only become slightly irritated and then heal up; in others, an imperfect attempt at the formation of pustules takes place; and in a third set, regular normal vaccine vesicles are formed, as after the first operation. Fresh fluid lymph should always be used for re-vaccination.

How far the susceptibility to be affected by re-vaccination is a test or indication of the susceptibility in the person of catching the variolous contagion—a question certainly of the very highest practical importance—has not yet been determined; although it seems probable that it is so. Dr. Jenner in one of his latest publications, distinctly states, "*if the constitution shews an insusceptibility of the one, it commonly does of the other.*"

11. The number and character of the *cicatrices* on the arm afford no means of judging whether the person is susceptible either of the vaccine or of the variolous inoculation.

12. Almost all the cases of very severe, certainly of fatal, small-pox after proper vaccination have occurred in persons upwards of fifteen or even twenty years of age.

13. No case of small-pox has, according to the German authorities, occurred in any re-vaccinated person.

14. We have no very accurate or sufficiently extensive data to ascertain the average frequency of secondary small-pox, after the natural disease or after inoculation with the variolous virus.

Dr. Baron, in his Life of Jenner, goes so far as to state that the number of cases of secondary small-pox is as great as that of small-pox after *perfect* vaccination. He appeals to a series of observations made at the Royal Military Asylum at Chelsea. From 1803 to 1833, it appears that 5592 children were admitted into that institution; of these 2532 were reported to have had small-pox, and 3060 to have been vaccinated.

The number who had small-pox after reputed small-pox was 26; and the number who had small-pox after vaccination was 24. The number vaccinated at the Asylum subsequent to admission was 628, of which number three only caught the small pox. The number who died of small-pox at the Asylum was four boys and one girl; of these five children, three had the disease after reputed small-pox, and two had neither been vaccinated nor undergone the small-pox before. (See Appendix to Report from Select Committee on the Vaccine Board.)

The statements in this document, if taken by itself, certainly warrant the conclusion which Dr. Baron has deduced; and it would seem to be confirmed by some other observations to which he alludes. Thus in the varioloid epidemic, which prevailed in Edinburgh in 1818-1819, a greater number of deaths occurred among those who had formerly suffered from small-pox, than in those who had been vaccinated; and Mr. Cross, in his excellent account of the epidemic at Norwich in 1819, alludes to three cases of fatal secondary small-pox, whereas two only of fatal small-pox after vaccination were heard of. It will be observed also that a statement, made by M. Dezeimeris respecting one of the recent epidemics in Norway, is in accordance with these observations.

Still it is right to bear in mind that we have much need of further authentic data, before we can justly arrive at any sound conclusions on this subject; more especially as we understand that the personal experience of Dr. Gregory is not in conformity with the statement made by Dr. Baron that "vaccination, when duly gone through, does certainly afford as complete immunity from subsequent attacks of small-pox, as that disease itself can do."

The number of cases of secondary variola, whether after inoculation or after the natural disease, which have come under Dr. Gregory's personal observation has been very few—not more than three or four unequivocal cases in all. He is of opinion that in very many reputed instances of secondary small-pox, the disease has been, *the second time*, either varicella, porrigo, or lichen varioloides.

15. Almost all the cases of unequivocal secondary small-pox have, according to the statements of some writers, occurred at from twenty to thirty years after the date of the first attack.

16. With respect to the effects of vaccination on persons, who have had the small-pox, it is stated by Dr. Heim of Wurtemburg, that of 297 such cases, the operation succeeded perfectly in 95, produced modified vesicles in 76, and failed altogether in 126—a proportion not dissimilar to what has been stated in some reports to have been the results of re-vaccination.

### Notice of the German Journal *Zeitschrift fur die Gesammte Medicin;* its Contents.

The eighth volume of the above periodical, edited by Drs. Fricke and Oppenheim, was recently sent to us for inspection; and we have been much pleased with the perusal.

It contains several original contributions from Professors Osiander, and Dieffenbach, and from Drs. Ruppius of Freiburg, Dubigk of Berlin, Nathan of Hamburg, Oppenheim, and Fricke.

The *second* part is occupied with analytic reviews of most of the important medical works, which have, within the last year or two, been published, not only in Germany, but also in Britain, France and Italy; such as Wardrop on Diseases of the Heart, Marshall Hall on the Nervous System, Guy's Hospital Reports, the Medico-Chirurgical Transactions, the Memoirs of the Royal Academy of Medicine at Paris, the Clinique Medicale of Bouillaud, Statistical Researches on Foundlings, Illegitimate Children and Orphans in France and other countries of Europe, by MM. Gaillard, Terme, Montfalcon, and Remache, Clinical History of the Cholera Morbus in Italy, by Drs. Renzi and Rotondo of Naples, &c. &c.

The *third* part comprises numerous valuable notices and extracts from almost all the leading medical journals of Europe, being similar to, but less complete and extensive than, the Periscopic Department of the Medico-Chirurgical Review; and the *concluding* part is occupied with miscellaneous notices, and intelligence about medical books, institutions, and so forth.

---

### Extract from M. Gendrin's Lectures on Diseases of the Heart.

"A vertical line, passing along the articulations of the cartilages of the left ribs with the sternum, represents the anterior edge of a vertical plane, which divides obliquely the heart into two unequal halves. The posterior edge of this plane will be found to correspond with the posterior edge of the inter-ventricular septum; the part of the heart situated to the right of the plane will comprehend the base of the right ventricle, and the right auricle; and the part to the left, will comprehend the inferior part of the right ventricle, the whole of the left ventricle, and also the left auricle.

The pulmonary artery may be considered as the axis of the heart: it springs from the base of this organ; and, passing somewhat upwards and sinistred, it crosses in front of the aorta.

If a horizontal line be drawn along the lower edge of the third rib, and a perpendicular line be then made to cross it, so that it passes over the sternal articulations of the left ribs, the point of intersection will be found very nearly to correspond with the origin of the pulmonary artery; and the horizontal line to indicate the loose edge of the pulmonary and aortic semilunar valves. The point of intersection of a second perpendicular line, drawn about half an inch exteriorly to the former one, points out the left segment of the pulmonary artery.

The aorta, on springing from the heart, passes from behind the pulmonary artery, somewhat to the right side, and reaching the median line, it is situated immediately behind or dorsed to the sternum.

If a needle be inserted midway between the second and third sterno-costal articulations, and pushed directly backwards, it will probably be found to pass through the point where the *ductus arteriosus* was given off from the pulmonary artery.

The apex of the heart corresponds—in the recumbent supine position of the body—to the interval between the fourth and fifth sterno-costal articulations, at about an inch and a half from the mesial line of the sternum.

Occasionally the apex of the heart is lower down than what we have now mentioned, and reaches to the interval between the cartilages of the fifth and sixth ribs. In general, it is a little higher up in women than in men.

The point of attachment of the pericardium to the large blood-vessels, which spring from the heart, is on a level with the arch of the aorta and behind the second sterno-costal cartilage. This fibrous sac is fixed inferiorly to the centre of the diaphragm, and extends to the left for about two inches: posteriorly, it rests, in an inclined position, upon the bodies of the sixth and seventh dorsal vertebræ.

We have already stated that a horizontal line, drawn along the inferior edge of the third left rib, corresponds to and indicates the position of the free edges of pulmonary and aortic semi-lunar valves: this line will be found also to pass on the level of the two auriculo-ventricular orifices.

*Sounds of the Heart.*—The heart is the seat of two principal movements, which follow each other alternately: the movement, which corresponds to the propulsion of the blood from the ventricles into the arteries, is the *systole;* and that, by which the empty ventricles become refilled with blood, constitutes the *diastole*.

These two movements of the heart coincide with two distinct sounds, appreciable by the ear when applied over the precordial region: the first sound corresponding with the systole, and therefore called *systolic*, and the second one with the diastole, and called the *diastolic*.

M. Gendrin subdivides each of these sounds and movements into three periods,—the *pre-systole*, the *systole*, and the *peri-systole;* and the *pre-diastole*, the *diastole*, and the *peri-diastole*—to indicate more exactly the consecutive changes immediately before, during, and after, the contraction and dilatation of the ventricles.*

To the alternate movements of the heart are to be attributed not only the coinciding sounds to which we have alluded, but also the phenomena of the arterial pulse. If a finger be applied over the *trajet* of an artery, we perceive a succession of beats, which are isochronous with the systole or contraction of the cardiac ventricles. This isochronism is not however quite perfect, except in those arteries which are very near to the heart: the interval between its systole and the arterial pulse becoming gradually greater and greater, according to the distance of the vessel from the centre of the circulation.

The arterial pulsation corresponds to and indicates the dilatation or diastole of the vessel, during which it becomes not only fuller, but is also somewhat lengthened, and its curvatures are increased.

The shock or force of the pulse is usually proportionate to the systolic impulsion of the heart: it is communicated along *de proche en proche comme par reptation*, becoming feebler as the arteries are more remote from the centre of the circulation. The arteries do not present, in a healthy state, a double beat like the heart. The swell or heaving, which they communicate to the finger, is produced by the diastole of the vessel; its systole being unattended with any impulse.

This absence of a systolic impulse is just what we might expect, when we consider that the arterial systole consists only in a return of the vessel to its normal calibre by the elastic power of its middle coat. We recognise then in the arteries, as well as in the heart itself, a diastolic and a systolic movement; and we

* We should deem this sub-division a very unnecessary and perplexing attempt at refinement.—*Rev.*

divide each of these movements into three periods, which we designate by the terms arterial *pre-diastole, diastole,* and *peri-diastole,* and arterial *pre-systole, systole,* and *peri-systole.*"—*Journal des Connoiss. Medic.*

---

### Opening between the Ventricles of the Heart in an Adult, without any Symptoms of Morbus Cœruleus.

A shoe-maker, 26 years of age and of a weak lymphatic constitution, had been subject to palpitations of the heart from his infancy, and had twice suffered from attacks of acute rheumatism. His present attack is pneumonia on the left side, the pericardium being probably affected at the same time. The attack proved fatal.

*Dissection.*—There were old and firm adhesions between the left pleuræ; the lung too, on this side, was partially hepatised and tuberculated.

On opening the pericardium, about two spoonfuls of serosity were found within. The heart was extremely large and hypertrophied, and was coated with patches of albuminous deposit on its outer surface. All the cavities and orifices were very wide and open. On carefully examining the ventricles, it was found that there was an opening immediately under the mitral and tricuspid valves, between the left and right sides. It was about an inch in diameter, with smooth and rounded edges,—indicating that it was an old formation. It was almost quite plugged up with the yellow coagulum, so frequently found after death within the heart. The *foramen ovale* was perfectly closed.

*Remarks.*—Cases similar to the preceding have been observed by Richerand and Meckel in persons of 40 and even 60 years or age.

MM. Louis and Bouillaud very justly remark, that in cases, where an abnormal communication exists between the two sides of the heart, either in the auricles or in the ventricles, there is often little or no cardiac distress, *provided* there be no contraction of any of the natural openings, or thickening or other lesion of the valves, to obstruct the free egress of the cavity from the different cavities.

The patency of the *foramen ovale* in adults, who perhaps have never suffered from heart disease, is well known to all pathologists. If, however, any disorder accompanied with difficulty of respiration, such as pneumonia, bronchitis, &c. supervene, such patients are very apt to sink, often quite unexpectedly, under the attack. It is a good rule therefore in practice, always to watch with double care any chest complaints in persons, who have been subject to palpitations or other disorder of the heart.—*L'Experience,* Jan. 1838.

---

### Cyanosis; Origin of the Aorta from the Right Ventricle, &c.

An infant, who had exhibited in a very marked degree the symptoms of *morbus cæruleus,* died in the second week after birth.

*Dissection.* The cerebral vessels were extremely gorged with blood: the lungs also were very highly congested, and did not crepitate firmly on pressure. The heart was very large, and shaped somewhat like that of the turtle. The right ventricle was highly muscular; from this cavity the aorta, as well as the pulmonary artery, was found to arise. The latter vessel, however, was nearly closed up; and no trace of the ductus arteriosus was visible.

The left ventricle was atrophied, and exhibited no appearance of aortic opening or of mitral valve. In the *septum cordis* there was a large round aperture, which permitted a very free communication between the two ventricles.—*Archives Generales.*

---

### IRREGULARITIES OF THE SUBCLAVIAN ARTERY AND VEIN.

The following case will illustrate what unexpected difficulties may arise in a case, where it is necessary to tie the subclavian artery.

A soldier received a stab in the axilla: a profuse hæmorrhage followed; and, although it seemed to be checked by pressure, Professor Dubreil did not hesitate at once to proceed to tie the subclavian artery. After the necessary incisions had been made, he felt for the tubercle of the first rib, and expected to find the artery where it emerges from between the *scaleni* muscles. But instead of the artery, the subclavian vein was in its place; and, as the wound was constantly filled with dark blood, he found it impracticable to search for the artery. He therefore determined, without loss of time, at once to tie the axillary artery between the deltoid and pectoral muscles. The patient died on the following day.

*Dissection.* On examining the wound it was found that the brachial artery, close to the point of its origin, had been penetrated. The ligature included the axillary artery alone, and none of the nerves were in any way injured. On tracing the subclavian vessels, the vein was found occupying the usual position of the artery between the anterior and middle *scaleni* muscles: it (the latter) was situated somewhat in front of and above the nerves, and was not easily exposed even in the dead subject. The distribution of the vessels on the left side was altogether normal.

The arterial irregularity, observed in the preceding case, has been noticed by several surgeons. Thus Velpeau and Blandin mention a case where the subclavian vein accompanied the artery between the *scaleni* muscles. Cruveilhier found both vessels situated in front of the *anterior scalenus* muscle, and in another case the *middle scalenus* passed between and separated the leash of the axillary nerves. Many other cases might be alluded to; but this is unnecessary.

The operating surgeon cannot be too well acquainted with all these irregularities in the distribution and relative arrangement of the great vascular trunks. —*Gazette Medicale.*

---

### PROFESSOR DIEFFENBACH ON THE ORTHOPÆDIC ESTABLISHMENTS IN PARIS.

The great progress, which Orthopædy has made of late years in France, has attracted the attention of most recent professional visitors to Paris. Indeed, after lithotrity, the treatment of deformities and contractions has become quite a favourite subject with many of the leading surgeons in that metropolis. Not many years ago, the management of such cases was not deemed worthy of the surgeon's attention; and it was therefore left to corset makers, and to others equally as uninformed, to do the best that they could to rectify, or at all events to conceal, the deformity. But now there are regular establishments, conducted by most able and intelligent men of education, in the neighbourhood of Paris, to which resort, from all parts of France, an immense number of persons who are afflicted with curvatures of the spine, contractions of the limbs and such-like maladies. The most celebrated of these are situated at Passy, a beautiful village in the immediate neighbourhood of Paris, and belong to M. Guerin, and to M. Bouvier. Both institutions are conducted with admirable skill, and afford to the invalids every advantage which the ingenuity and experience of these gentlemen can afford. The arrangements for gymnastic exercises, for the use of extending machinery and bandages, for the improvement of the general health of the patients, and at the same time for the recreation and instruction of the mind, are most complete in every particular.

In cases of curved spine, the extension is effected by means of a peculiarly constructed bed to which the patient is secured, and the length of which may then be increased or diminished as the surgeon may require. The one used and recommended by M. Guerin consists of four different segments or smaller beds, which can be separated from each other or screwed together at pleasure. By moving one or more of the pieces to either side, after the patient has been secured by the bandages and straps, a certain lateral direction may be given to the extending force, so as to draw the spine either to the right or to the left hand, according to the inclination of the curvature. The peculiarity of M. Guerin's extending bed is its being composed of several distinct pieces. The entire establishment of this gentlemn at Passy deserves the highest praise. The great Monthyon prize was some years ago awarded to him by the Royal Academy at Paris, for the various improvements which he had introduced into the scientific practice of Orthopædy. His museum contains numerous specimens of comparative as well as human anatomy, and also a most instructive collection of Paris-plaster casts of the various forms of distortion and irregularity of the spine and joints, at various periods of their treatment.

The employment of gymnastic exercises holds a prominent place in the Orthopædic practice of M. Guerin. In the great hall of his establishment are to be seen all sorts of machines and apparatuses for the use of the inmates. Swimming and other exercises, in which the body lies as in swimming, are particularly recommended both by M. Guerin and by M. Bouvier. Another exercise, which is very generally practised by young invalids, is going on tall crutches. "I was," says Professor Dieffenbach, "quite astonished, upon entering the gardens, to see a number of French girls moving about with surprising agility on tall crutches, like so many kangaroos with their short arms and long legs." The staves of the crutches are very tall, so that when the invalids stop, their feet do not touch the ground, but are suspended, *schwebend in der luft,* half an ell above it, and the body is supported by the arms.

Professor Dieffenbach seems to approve of this mode of exercise, and recommends it to his countrymen for imitation. He mentions with praise the collection of casts and other illustrative preparations in the museum of M. Bouvier, and alludes in particular to one cast, which M. Bouvier directed his attention to, as being quite "une moule historique." Its history is rather curious and may be instructive as well as interesting to the reader.

An orthopædist of some eminence in Paris announced, some time ago, to the Institute of France, that he had discovered a method of removing curvatures of the spine in a very short period of time; and, with the view of convincing the members of the truth of his claim, he brought before their notice a young woman in whom there was a very marked irregularity of the spine, which he undertook to rectify in the course of a few weeks. A commission was appointed to investigate the subject; Paris-plaster casts were taken of the patient's back; and every other means to arrive at the truth were adopted.

Within a very short time, the girl was again presented before the Institute, and now not the slightest trace of deformity was visible. The commissioners were quite satisfied of the identity of the individual, and could not help expressing their astonishment at the extraordinary rapidity of the cure: it seemed to them quite miraculous. M. Guerin however suspected that there must be some deception, and that the case was in all probability one of simulation.

He soon convinced the commissioners of the truth of his suspicions, by proving to them that, although the line of the spinal column seemed to be quite as much deformed in the simulated as in the genuine case of the disease, the muscles of the back were at the same time dislocated in the latter, but not in the former, instance. By attending therefore to the condition of the spinal muscles in a suspected case, we may generally detect when a fraud is attempted to be played upon our judgment.

The zeal, with which M. Guerin exposed the impudence of the above trick, so irritated the impostor that he brought an action at law against his adversary; and, although all right and equity were on the side of M. Guerin, he was amerced in a heavy penalty for the injury he had done to the plaintiff's character.

Professor Dieffenbach pays a high compliment of admiration to M. Bouvier for the pleasure and instruction he had received from visiting his orthopædic establishment; and he alludes with great satisfaction to his having met there some of the ablest professional men in Paris, such as the two Larreys, father and son, Marjolin, Le-roy, and many others. The second prize of 2000 francs has been awarded by the Institute to M. Bouvier for the various improvements which he has introduced.

For some time past, he has had the charge of all cases of deformity in the Hôtel Dieu, and also in the Hôpital des Enfans Malades; and at these two great institutions, the medical students have an opportunity of acquiring a scientific knowledge of Orthopædy.

Besides the establishments noticed above, there has been for some time a sort of ambulatory infirmary for the treatment of deformities, in Paris; this is under the management of MM. Bouvier and Duval. At a stated hour each day a great number of cases, among children chiefly, come for advice, bandages, and so forth—all which are given gratuitously.

M. Duval attends to the cases of club-foot, and M. Bouvier to those of spinal deformity. In lateral curvatures of the back among the poor, and those who cannot be treated at home with the extending bed, M. Bouvier employs the girdle or cincture invented by M. Hossard (ceinture à levier ou inclinatoire), by means of which the depressed shoulder is elevated, and the spine is inclined somewhat over to the side opposite to that of the curvature.

Many of the cases of club-foot are treated by section of the tendo-Achillis, as recommended by M. Stromeyer.—*Zeitschrift fur die ges: Med.*

---

### CONCOURS FOR THE CHAIR OF ORGANIC CHEMISTRY AT PARIS.

The following brief notice of the *tests* to which the rival candidates *were submitted*, at the late *concours* for the chair of organic chemistry and pharmacology will possibly be interesting to the English reader. The election by *concours* may, we will allow, be attended with some disadvantages, as it has been often remarked that professional and scientific men are generally the least impartial judges of professional and scientific merit; but that there are corresponding and, we may add, overbalancing benefits from its adoption, cannot be well disputed.

The *first* examination was altogether occupied with the preparing of a written thesis upon the organic alkalis.

At the *second* examination, the candidates had to discourse orally, after twenty-four hours' preparation, upon a given subject. M. Baudrimont upon the chemical and pharmaceutical relations of alcohol; M. Bouchardat upon those of the essential oils; M. Bussy upon those of adipose and fatty matters; and M. Dumas upon those of sugar.

At the *third* examination—which also was an oral one, after two hours' preparation, upon a subject determined by lot—MM. Baudrimont and Bouchardat had to treat of the properties of albumen and gelatine, and MM. Bussy and Dumas upon those of milk.

At the *fourth* and last examination, the candidates were required to defend their respective printed theses. M. Bussy on the urine and its changes in diseases; M. Dumas upon the influence of heat on organic bodies, and its employment in pharmaceutical preparations; M. Bouchardat on the blood; and M. Baudrimont on the present state of organic chemistry.

Each candidate gave convincing proof of great knowledge and acquirements; but as the superiority of M. Dumas was acknowledged by all, he was unanimously elected to the professional chair.

---

### Treatment of Diptherite, or Croupy Inflammation of the Mouth.

Dr. Ruppius, of Freiburg, very justly remarks, that the morbid action or process in this disease—first accurately described by M. Bretonneau of Tours—is essentially the same as characterises the well-known disease of *Croup* or *Cynanche Trachealis*. In both, the inflammation of the affected mucous tissue has a very marked tendency to cause an exudation of a membraniform lymph on its surface: in the one, this phenomenon is usually limited to the trachea and lower part of the larynx; while in the other, it is observed chiefly on the velum palati, the tonsils, and back parts of the fauces.

*Diptherite* usually commences with the ordinary symptoms of *Cynanche*. There is some difficulty experienced in deglutition, arising from the swelling of the tonsils; the act of inspiration is attended with a slight snoring noise; the voice becomes sharper and shriller; and the system is generally more or less feverish. If the fauces be examined, the surface, especially that of the tonsils and uvula, is observed to be of a purplish red colour.

When the exudation commences, a troublesome cough is apt to come on, the breathing becomes more and more difficult, and the patient is more restless and agitated. If the morbid action extends to the opening of the larynx, the symptoms become much more alarming, and the local distress assumes much the same character as is present in croup. Such cases usually prove fatal.

The treatment of this disease is to be conducted on the same principles as that of croup—bloodletting, local and general, in the early stages, and the use of calomel and of antimonials in such doses as to make an impression on the system. Along with the adoption of these means, Dr. Ruppius strongly recommends the local application of the nitrate of silver to all the parts of the fauces, which are not invested with the exuded lymph. The marked efficacy of this treatment, in arresting and in modifying the morbid action, is often very gratifying. There is no objection to the applying the nitrate to the parts which are covered with the lymph; but it seems to be generally useless.—*Zeitschrift fur die ges. Med.*

---

### Professor Osiander on Puerperal Fever.

Professor Osiander premises his remarks, by observing, that the term 'Puerperal, or Child-bed Fever,'—although not scientific, nor consonant to the nomenclature of other febrile diseases—cannot be well replaced by any other which has been proposed, such as *peritonitis puerperalis, metro-peritonitis,* &c. The peritoneum and the uterus may be quite free from inflammation, and yet the patient may die from puerperal fever.

Often the disease commences as an attack of meningitis, or of erysipelas, or of rheumatic swelling of the joints, &c. and, under any of these forms, it speedily proves as dangerous and alarming as when the uterus and peritoneum are primarily affected. For this reason, Professor Osiander prefers to retain the old generic term of Puerperal fever, and of distinguishing its various species or forms in the following manner, according to the seat and character of the predominant local mischief.

1. Puerperal fever with peritonitis.
2. .. .. .. hysteritis and peritonitis.
3. .. .. .. meningitis.
4. .. .. .. pneumonia.
5. .. .. .. miliary fever.
6. .. .. .. erysipelas.
7. .. .. .. arthritic swelling.
8. .. .. .. milk abscess.
9. .. .. .. gangrenous inflammation of the external and internal generative organs, (metritis gangrenosa).
10. .. .. .. typhus fever.

The last-mentioned variety includes, according to this tabular arrangement, all those cases of child-bed fever, in which there is a suppurative inflammation of the uterine and adjacent veins. It is by far the most formidable variety of the disease, and is that which usually prevails epidemically at certain seasons, more especially in large institutions. To apply the term *peritonitis* or *metro-peritonitis* to it, is not only quite incorrect—seeing that often no genuine traces of peritoneal inflammation are discoverable on dissection—but is likewise most seriously hurtful, in consequence of the erroneous treatment which will necessarily be recommended.

An impure condition of the atmosphere—attributable very often to an over-crowded state of the wards in a lying-in establishment—is unquestionably one of the most frequent causes of puerperal typhus. That this form of the disease is of a miasmatic origin, and is communicable from one patient to another, cannot be well disputed; and it therefore becomes the duty of the physician and nurse to use all precautionary means to prevent the dissemination of the miasm, by changing their own garments frequently, washing their hands, &c. as well as by the employment of fumigations and other well-known means.

Much may be done in the way of *prophylaxis* of this disease, but very little in the *treatment* of it, when it is once fairly established. There is perhaps no form of fever so little under the control of medicine as puerperal fever: it is only in the precursory and very early stages of the disease that the healing art can be of any avail.

According to the researches of Osiander, inflammation and suppuration of the uterine veins is by no means so generally present, as some authors might lead us to suppose.

Inflammation of the lymphatic vessels seems to be of much more frequent occurrence when the peritoneum has been inflamed, than phlebitis of the uterine veins.

The latter is however by far the most serious affection of the two. Abscesses of the liver, of the lungs, and of the muscles and joints, are not uncommon sequelæ of uterine phlebitis, when the patient has survived the early stage of the disease.

The following few cases illustrate some of the most generally observed characters of puerperal typhus.

CASE 1.—*Puerperal Typhus, fatal in 24 hours, with Peritonitis, and Suppuration in the Uterine Lymphatics.*

A woman, 27 years of age, was delivered in the Maternité Hospital, at a time when child-bed fever was very prevalent. For three days subsequently she went on very well; but, on the fourth day, she began to complain of pain in the hypogastric region. The pulse, at this time, was frequent, but not full or hard; and the patient was troubled with diarrhœa and tendency to vomiting. *An unfavourable prognosis was formed, in consequence of the great frequency*

*of the pulse.* Thirty leeches were applied to the abdomen, and an ipecacuan emetic was administered; an ounce of mercurial ointment was rubbed in upon the inside of the thighs, and a sinapised hip-bath was used.

In the evening, the abdominal pain was less severe, but the pulse was still rapid and small: the diarrhœa and nausea continued.

Next day, the abdomen was tympanitic, and very painful; the breathing was hurried and uneasy; but there was no delirium, or mental confusion. Sinapisms and friction with the mercurial ointment were continued; but the patient died in the course of the night.

*Dissection.* The abdomen was found to contain a quantity of muddy serum, blended with numerous flocculi of albuminous matter—the result, no doubt, of peritoneal inflammation. Beneath the peritoneum, in the left iliac fossa, there was a purulent infiltration into the cellular tissue: this extended upwards as high as the kidney. The lymphatic vessels of the broad ligaments were filled with pus; but the uterine veins seemed to be entirely sound.

Case 2.—*Puerperal Typhus, with Suppurative Inflammation in the Sub-peritoneal Cellular Substance, and in the Uterine Lymphatics.*

A woman was seized, on the second evening after a fortunate delivery, with shivering and abominal pain. Upwards of fifty leeches were applied on the hypogastrium; and fomentations and other means were used. But on the following day the patient was moribund, and she died in the course of the evening: the abdomen had become exceedingly inflated.

*Dissection.*—The abdominal cavity contained some reddish-coloured serum; and between the uterus and rectum a small quantity of consistent pus was found. In the region of the cæcum some purulent matter existed behind the peritoneum. The lymphatic vessels, which accompany the spermatic veins, were full of matter.

*Remark.*—We are inclined to believe that the inflammation must have, in this case, commenced previously to delivery.

Case 3.—*Puerperal Typhus fatal in fifteen hours after Delivery; Peritonitis and Suppuration of the Lymphatics; softening and Melanosis of the Lungs.*

A young woman was admitted, in the eighth month of her pregnancy, into the Lying-in Hospital on the 17th of June, at a time when the child-bed fever was very prevalent. On the third day afterwards, she experienced abdominal pain, and feverish excitement.

On the 25th, she was delivered of a dead child; and, on the following day, she died.

*Dissection.*—The abdomen contained a large quantity of white matter; and the lymphatic vessels on the sides of the uterus were filled with pus. The inferior lobe of the left lung was softened, and of a black colour.

Case 4.—*Puerperal Typhus, fatal in* 50 *hours; Peritonitis, and Suppuration of the Lymphatics.*

A middle-aged woman was seized, on the evening after her confinement, with shivering. Next morning, she had abdominal pains; the pulse was exceedingly quickened, and very easily compressed; there was slight diarrhœa; the lochia were not much affected. *Osiander* formed a very unfavourable prognosis of the case, in consequence of the extreme rapidity and feebleness of the pulse: he had not seen one patient recover in whom this state of the circulation existed. As he had found evil only to result from sanguineous depletion in any form, and from the use of emetics, as recommended by some physicians, he contented himself with recommending the application of poultices to the abdomen, and of camphor ointment around the pubes and to the thighs, and of warm injections into the vagina. By the use of these means, the patient was somewhat easier towards evening. Next day the abdominal pain was gone, and the pulse was not quite so rapid: five grains of ipecacuan were ordered to be given every four or five hours; and the warm-baths were to be continued.

But soon afterwards she became gradually worse, and she died 68 hours after delivery, and about 50 after the commencement of the feverish symptoms.

*Dissection.*—The abdomen contained some purulent serosity; and pus was found in the uterine lymphatics, in the Fallopian tubes, and in the cellular tissue of the pelvis.

*General Reflections.*—Professor Osiander closes his memoir with a few remarks on the probable exciting, or predisposing, causes of child-bed fever. He insists particularly on the pernicious influence of cold air on women in the puerperal state; and he attributes the lamentable frequency and fatality of the disease, in the large lying-in institutions in Paris and Vienna, to the continual ventilation of the wards.

He mentions, that whereas it is almost constantly more or less prevalent in these hospitals, it is comparatively rare in the Maternité at Gottingen, whose wards are small, never crowded, and uniformly kept in a state of pleasant warmth. He is therefore of opinion that, if in lying-in establishments more attention was paid to the warming of the wards, and to avoid the crowding of many patients together, the epidemics of puerperal fever would be much less frequent and fatal.

He has never seen any good effects from the administration of calomel and active purgatives in this disease. He recommends the use of saline and tonic medicines; and he mentions that the application of large mustard poultices to the mammæ—so as to excite a powerful revulsion from the uterus—has in several cases seemed to act more beneficially than any other means, which he has employed.—*Zeitschrift fur die ges. Med.*

---

### Clinique of M. Bricheteau at the Hôpital Necker.

Case 1.—*Typhus—Sudden Death—Softening and incomplete Perforation of the Stomach.*—A girl, 17 years of age, was admitted into the Hôpital Necker on the 12th of April, with symptoms of typhus fever.

On the third day after her admission, while she was under a course of laxative medicines, she was seized with a sharp pain in the abdomen, in consequence of which the medicines were discontinued. The pain ceased by the application of leeches; but soon afterwards, this remission was followed by furious delirium, and this by stupor and ultimately complete coma; which—notwithstanding the use of blisters, ice to the head, sedatives, &c.—continued till she died on the sixth day.

*Dissection.*—The *brain* was slightly and only partially congested. The *lungs* and the *heart* seemed to be healthy. The mucous membrane of the *stomach* was softened and *worn-away*, as it were, to its peritoneal coat, in the neighbourhood of the cardiac orifice. Even the peritoneal coat was *usé;* for it had become as thin as gauze-paper; but this slight thickness had proved sufficient to prevent abdominal extravasation.

*Remarks.*—M. Bricheteau confessed his great surprise at the phenomena of the preceding case. From the violence of the cerebral symptoms he had prognosticated the presence of meningitis, and had expected to find upon dissection very decided marks of inflammatory action. Judge then of my surprise, he said, at finding the encephalon almost quite healthy.

The lesion too, of the stomach, was equally unlooked for. That it must have existed for a length of time, before the girl's health was at all affected, is more than probable. We see here an apt proof of a position, which ought to be well remembered by every medical man—*that serious organic mischief may exist without being attended with any disturbance of the health.* In the present case there had been no epigastric suffering until the attack of pain, after the admission of the patient into the hospital.

Are we to attribute the cerebral symptoms to sympathy with the morbid state of the stomach ? This is a question most difficult to answer ; and indeed the case altogether is a very puzzling one. The majority of the symptoms indicated a lesion of the encephalon ; and yet the pathological changes existed altogether in the stomach.*

Case 2.—*Epilepsy ; Sudden Death during a Paroxysm ; Attenuation of the Membranes and Perforation of the Stomach.*

A young woman, of a very corpulent frame, was brought to the hospital while under an attack of epilepsy. The fits returned upwards of twenty times in the course of the day, and were followed by a profound coma, the precursor of death. The patient was *enceinte* for the first time ; and it was stated that chagrin, from having been cruelly deserted, had probably been the cause of the attack.

*Dissection.*—The substance of the brain was found somewhat softened. A dark-coloured and rather fœtid fluid was found effused within the abdominal cavity ; but the viscera did not seem to be inflamed : the fluid had escaped from numerous perforations in the great *cul-de-sac* of the stomach. These perforations, when examined, presented the aspect of having been made with an *emporte-piece*, and exhibited no traces of inflammation on their edges. The mucous membrane of the stomach was much softened and attenuated ; and here and there it seemed to be entirely wanting. The uterus contained a well-formed fœtus, six months old.

*Remarks.*—Two opinions as to the cause of the perforations of the stomach were entertained. Some attributed them to the corroding action of the dark fluid which we have spoken of ; while M. Bricheteau considered it more probable that the violent contractions of the stomach during the epileptic paroxysm had induced a rupture of its coats, and that this lesion was the cause of the death. That the death had been sudden was apparent from the entire absence of all inflammatory signs on the abdominal viscera.

A third opinion might be hazarded on this subject ; for we might suppose that the perforations in the stomach were *post-mortem* phenomena. The experiments of Dr. Carswell of London have clearly shewed how often such a cadaveric lesion occurs in the lower animals, that have been suddenly killed.—*La Lançette Française.*

### Use of Caustic Issues in Phthisis Pulmonalis.

*M. Bricheteau* has for some years past been in the habit of using, with very decided advantage, caustic issues, formed immediately below one or both clavicles, in cases of pulmonary consumption ; even when the disease is far advanced, and auscultation has indicated the existence of tuberculous caverns in the lungs. That a powerful local derivative, like an issue, may have a decided influence in

* The cerebral symptoms are doubtless to be regarded as part of the phenomena of the existing typhus fever, and cannot well be attributed to the mere sympathy of the brain with the stomach. The absence of necroscopic changes in such cases is not of unfrequent occurrence.—*Rev.*

arresting at least the progress of morbid action, however serious, in an internal viscus, is well known to every medical man; and that in many cases it has this effect on the softening and ulceration of tuberculated lungs cannot, in M. Bricheteau's opinion, be gainsayed by any unprejudiced observer of his practice at the Hôpital Necker. Even where an absolute cure is not obtained, a great mitigation, and often a marked retardation, of the disease follows the establishment of caustic issues below the clavicles—provided always the rest of the treatment be at the same time judicious and appropriate.

We shall very briefly mention the histories of two cases recently treated in the hospital.

A young married woman was admitted in the following state on the 6th of June, 1837.

She was distressed with cough, puriform expectoration, copious sweats, and vomiting after the fits of coughing: there was a sharp stitchy pain felt over the right side of the chest. On examining the chest with the stethoscope a distinct gurgling sound was audible beneath the right clavicle; the respiration was cavernous behind; and these two symptoms became more *prononcés*, when the patient coughed; there was considerable dulness on percussion beneath the right clavicle. The patient was so weak that she could not walk about.

A large caustic issue was established immediately under the right clavicle, and demulcent medicines and diet were prescribed.

This treatment was persevered in for six or seven weeks; and by that time most of the unfavourable symptoms had disappeared, and the woman began to recover her flesh and strength. Ultimately she did well.

*Case* 2.—A middle-aged woman had, after repeated attacks of hemoptysis, become affected with all the usual symptoms of pulmonary consumption—cough, copious puriform expectoration, night sweats, and diarrhœa. She was considered by the physicians of La Charité hospital to be decidedly phthisical. Under the use of a large caustic issue beneath the right clavicle, and appropriate attention to the most troublesome existing symptoms, this woman regained her health so well, that in the course of two months she was able to leave the hospital, and soon after resumed her occupation of a washerwoman.

At a subsequent period this woman was admitted for a syphilitic affection. Her pulmonary symptoms had not returned. On auscultating the chest, the respiratory murmur was found to be very feeble under the right clavicle, and there was considerable dulness on percussion over that point. Posteriorly the sounds indicated the adhesion of the pulmonary pleura to the ribs.

*Remarks.*—The treatment recommended by M. Bricheteau—who is by no means, like some of his *confreres* in the French metropolis, a quick thinker and a rapid writer—is judicious, and deserves to be more generally tried than it has been.

The use of caustic issues in arresting deep-seated organic mischief is perhaps the most efficacious of all remedies, provided always an appropriate constitutional treatment is followed out at the same time. We have reason to believe that the remedial powers of iodine in the *early* stages of tuberculous disease have not been sufficiently appreciated. We speak not of Sir C. Scudamore's plan of inhalation, which is of very questionable utility, but of the internal administration of some of the milder salts of iodine in combination with vegetable bitters and demulcents.

Let it not be supposed that we are one of the believers in the *curability of consumption,* in the sense that some unprincipled members of the profession make a public parade of. We only wish to remind our readers that tuberculous, like every other morbid, formation is capable, in many instances, of being arrested or retarded in its progress; and that it is quite possible that Nature will, under these circumstances, sometimes effect a permanent cure.—*Rev.*

---

### M. Bricheteau on Typhus Fever.

*M. Bricheteau* commences his remarks by observing that no disease exhibits so many and so important different shades or types as Typhus; and therefore, that no single description, and no exclusive system of treatment, can possibly be universally applicable. Almost every epidemic has some peculiar features which modify the generic character of the disease, and which must necessarily influence the treatment by the unprejudiced physician. It is needless to tell us that all Typhoid fevers have their primary seat in a morbid state of the intestinal mucous follicles. This doctrine may, or it may not, be strictly true: we (M. Bricheteau) are rather inclined to assent to it. But however this may be, every one, who is acquainted with the history of past, and with the characters of recent, epidemics of typhus fever, will admit that at different seasons and in different places the disease exhibits very different phenomena, and requires very different remedies.

In one epidemic, the fever is almost uniformly petechial; in another, it is accompanied with vomiting and diarrhœa; in a third it is complicated with bronchitic symptoms; and in a fourth with *ataxia* and extreme disorder of the nervous system.

There cannot be doubt that the disease, as we have observed it of late years, is considerably different from that which came under the notice of Pinel, Petit, Corvisart, &c.

The conclusion is therefore forced upon us that Typhus—and we add other forms of—fever, without being precisely altered in its essential nature, exhibits, at certain periods, very marked modifications of character, and requires very different, nay opposite, modes of treatment; and all this quite independently of such differences as are attributable to peculiarity of constitution, mode of living, general state of health, the influence of remedies, and so forth.

This most important maxim was forcibly insisted upon by Sydenham who, in his work on acute diseases, most distinctly states that epidemics of continued fever, although resembling each other in respect to many of their symptoms and general characters, are often very different from each other; for a mode of treatment, which is useful in one, may be decidedly pernicious in another.

How then can any unprejudiced physician persist in recommending any exclusive or universal mode of treatment? Yet such is the case, even in the present day; and perhaps nowhere more dogmatically and more absolutely than in this great metropolis (Paris).

The purgative treatment is that which M. Bricheteau generally adopts, and which—allowing for individual as well as for climatic differences—he decidedly prefers to any other. Not only are the bowels thus relieved from all acrid offending matters, such as bile, mucosities, and fæculent matter, but there is also, at the same time, a powerful derivative action induced on the cerebral and thoracic organs.

The beneficial action of purgatives in typhoid fever has been explained in three different ways;—by modifying, after the manner of other contra-stimulants in cases of angina, aphthæ, ophthalmia, and chronic exanthemata, the mucous membrane of the bowels and the follicular glands—by simply evacuating from the bowels all offending and acrid matters, such as bile, mucosity, and fæces—or, lastly, by acting indirectly upon the circulating fluid, in consequence of the discharge of the serum, and perhaps also of any noxious miasmatic matters introduced into the system, along with the intestinal evacuations.

M. Bricheteau is inclined to adopt the first of these opinions; and M. Bouillaud seems to favour the last.

But in whatever manner we choose to explain the action of purgatives in typhus fever, there cannot be a doubt, says the former of these gentlemen, that they are very often highly useful in cases, the symptoms of which might seem to contra-indicate their use—for example, when the tongue is hot and dry, the epigastrium and abdomen tender and painful, the skin parched, and the thirst and fever intense.* In very severe cases, however, where we have reason to suppose that the intestinal ulcerations are far advanced, and in which, therfore, we have to fear the occurrence of perforation of the bowels and the subsequent discharge of their contents into the abdomen, the use of purgatives ought probably to be abstained from; or, at least, to be adopted with extreme caution.† M. Bricheteau is quite opposed to the system of repeated bloodlettings so perseveringly insisted upon by M. Bouillaud.

As an appropriate pendant to these remarks on typhus fever, we shall now relate two very remarkable cases of a *hæmorrhagic diathesis*, arising probably from a dissolved state of the blood, such as occurs in certain forms of putrid fever.

*Case* 1.—A mason, 21 years of age, had but imperfectly recovered from an attack of typhus fever, when he was admitted into the Hôpital Necker. He was extremely weak, had a troublesome cough, and flying pains in different parts of the body; the abdomen was tense and uneasy; the bowels were confined; and the system was feverish.

His face became puffy and livid; the eyelids were infiltrated, and the conjunctivæ exsuded blood. From the mouth also there was a sanguineous exsudation; and several coagula were evacuated from the bowels. He rapidly sunk, and died.

*Dissection.*—About three glassfuls of bloody serum were found in the abdominal cavity. The internal surface of the bowels was healthy as low down as the neighbourhood of the ileo-cæcal valve, where there were observed several elliptic spots (plaques), the effects of previous ulcerative inflammation.

The pleuræ, and also the pericardium, contained a quantity of sanguineous fluid. The heart was sound; but there was no blood in any of its cavities. The tissue of the lungs was very soft and lacerable.

The serous membrane of the brain contained in its meshes a quantity of gelatinous, partially coagulated, bloody fluid; and blood was found within the cerebral ventricles. The substance of the brain was exceedingly soft. Blood was found within the knee-joints, and also within the tunica vaginalis. The other serous cavities were not opened.

---

* M. Bricheteau seems, in this passage, anxious to expose the errors of the Broussaian school; one of the tenets of which has been to eschew the use of purgatives in typhus fever.

† M. Bricheteau closes his remarks on the treatment of typhus, without even adverting to the use of any other remedies except purgatives. Such is the besetting sin of all *doctrinaires* in our profession: they form theories, and on these they build their practice. Are saline diaphoretics and refrigerants of no utility? are emetics never serviceable? is cold or tepid spunging of the surface of no avail? and is it not necessary in many cases to have recourse, even from the first, to stimulants, as wine, brandy, and ammonia? If the typhus fever of Paris is at all like that which we see in London, we have little hesitation in saying that neither the purgative, nor any other, plan of medication is suited to all cases of the disease; the treatment must be varied, according to the peculiarity of each patient's constitution, as well as of the prevailing type of the epidemic. —*(Rev.)*

*Case* 2.—A labouring man died, after being a few days ill, with all the symptoms of hæmorrhagic Purpura.

*Dissection.*—The peritoneum and also the pleuræ were spotted over with numerous petechiæ; and in many places, where there is much cellular substance, there were immense ecchymoses.

About six ounces of blood, mixed with a small quantity of serum, were found in the left pleura. Both lungs were partially infiltrated with blood. The pericardium, and also the surface of the heart, was spotted with ecchymoses: the tissue of the latter was remarkably soft, and very easily torn. The inner surface of the stomach was here and there ecchymosed; and was so little consistent, that it might be scraped off with the back of the scalpel. Almost the whole extent of the intestinal mucous surface was in a similar condition. The substance of the spleen, and also of many of the muscles of the body, was soft and gorged with blood.

The head was not opened.

M. Bricheteau says, that he is inclined to attribute such cases, as the preceding, rather to a primary affection of the solids, which diminishes their normal adhesion and resistance, than to any degeneration of the blood.—*La Lançette Française.*

---

### Cantharides proved to be a Contra-Stimulant, or Antiphlogistic Remedy: its Depressing Action on the Heart, &c.: its great Utility in Inflammatory Diseases.

The following *really* practical observations are exceedingly interesting, and well deserve the notice of medical men. Most readers will probably be surprized at first with some of the positions: but as they profess to be drawn from, and based on, actual experiments, both in health and in disease, we must not allow our *British* prejudices to blind our judgments.

Without any further premise, we shall now give the substance of the paper by Dr. Giocomini—one of the physicians of the hospital at *Padua*—and leave our readers to judge of its value for themselves.—*(Rev.)*

### *First Series of Experiments.*

Nine medical students took, each, a pill, containing one grain of powdered cantharides, at eight o'clock in the morning.

In the course of two hours, the pulse in every one of them had fallen in frequency—from two to fourteen beats in the minute. In five the urinary secretion was increased.

A second pill was now taken; and again another at noon. All of them now complained of heat in passing their urine, which continued to be very abundant, and of general weakness and tendency to profuse perspiration, although the weather was not hot. Some suffered from colic pains, tenesmus, and a troublesome itching about the anus. In all, the pulse continued to be considerably under the natural standard. None of them slept soundly during the following night, in consequence of the heat in the urethra, and the prostration of the system; but, by the next morning, they had all recovered from the effects of the medicine. One, who had for some time been annoyed with chronic ophthalmia, found himself entirely relieved from it.

### *Second Series of Experiments.*

To each of seven youths a pill, containing a grain and a half of the cantharides, was given. Two hours afterwards, the pulse, in five of them, had fallen from five to fifteen beats in the minute; in the remaining two, the pulse was quickened by two beats, but was considerably softer than it had been. Another pill was now given; and a third at noon. At three o'clock, the pulse in all had become slower than before; all complained of extreme exhaustion and of copious perspiration: two only suffered from urinary distress, the rest having drunk very freely of mucilaginous drinks during the day. Next day, all the symptoms induced had passed away, with the exception of the general languor. One of the youths, who had been habitually troubled with palpitations of the heart, was quite relieved from them.

*Third Series of Experiments.*

Six students tried the effects of *cantharidine.* Each of them took a fourth of a grain, and repeated the dose, at intervals of two hours, for three times. Demulcent drinks were used freely at the same time. In all of them, there was a progressive retardation of the pulse; the *maximum* of this retardation was 22 beats in the minute. All of them suffered from general languor and powerlessness; from vertigo, and tremblings of the limbs; from loss of appetite; and from excessive perspirations. The sense of burning along the urethra was present in all; but considerable in one only. In some there was a troublesome diarrhœa. The general exhaustion continued, in a greater or less degree during the whole of the following day. The symptoms were found to be most effectually relieved by drinking freely of wine and of other spirituous beverages; which, although taken by some in large quantities, did not induce any tendency to intoxication.

Before quitting the details of these experiments, we should mention that the symptoms of poisoning were so alarming in one of the youths, that his case deserves to be noticed by itself.

Canton Bartolomeo, 23 years of age, had submitted himself to the three successive series of experiments. Shortly after having taken the last dose of the cantharidine, his pulse became slow, his limbs tottering; and vertigo and confusion of mind came on. The urine, which at first was very abundant, became suddenly altogether suppressed, and the patient complained of intense pain in the region of the kidneys.

The prostration increased; the limbs became quite powerless and cold; and the pulse was thready and exceedingly slow, not exceeding 30 beats in the minute.

Dr. Giocomini finding that the wine, which he gave him pretty freely, was vomited, began to ply him with *rum,* in such quickly repeated doses, that in the course of little more than one hour, he took upwards of half a pint of it. The symptoms of poisoning gradually passed away, and the patient did not feel himself at all inconvenienced by the quantity of spirit, which had been given him.

From the series of experiments which we have now recorded, two very important conclusions may be derived with respect to the action of cantharides on the system.

1. That it is contra-stimulant, hypo-sthenic or depressing; its operation being exerted chiefly on the circulating system.*

2. That its most effectual antidotes are stimulating substances, such as alcoholic liquors, ammonia, laudanum, æther, &c. It is quite a mistake to suppose that camphor is an antidote to cantharides—both are contra-stimulants.

The therapeutic action of cantharides in disease is quite accordant with these propositions. *Signors Borda* and *Rasori* have treated many cases of acute

---

* Dr. Giocomini is inclined to believe that cantharides, even when used locally as a blister, has a contra-stimulant or antiphlogistic operation; and therefore that other vesicants, such as boiling water, strong ammonia, &c., cannot be so serviceable in the treatment of many complaints, as the blisters made with the Spanish fly.

pneumonia with large doses of cantharides alone; and they have derived the same results from them as from the use either of copious bloodletting, or of large doses of antimony or of digitalis. The *tolerance* of large doses of cantharides, in acute or *hypersthenic* diseases, is quite as remarkable as, it is well known, is the case with these last-named remedies under similar circumstances. What would inevitably produce symptoms of poisoning in a state of health, proves to be only a salutary medicine when the system is *hypersthenic*.

The degree or extent of *tolerance*, by the system, of cantharides or any other contra-stimulant furnishes a most useful guide to the practitioner in the treatment of acute or inflammatory diseases; as it has been found that the *tolerance* is almost always proportionate to the violence of the disorder, and becomes less and less, as it abates.

S. *Larber de Bassano* has employed cantharides in the treatment of all inflammatory diseases without exception; and he reports most favorably of the practice. The action of the heart and sanguiferous system is rapidly abated, and a *crisis* by urine or by perspiration soon follows. Its irritating effect on the bladder may be counteracted by the copious use of demulcent drinks.

The following case of *pleuritis* treated with cantharides by Dr. *Giocomini* will serve as an example of the practice. A robust, middle-aged man, was seized with the usual symptoms of pleurisy on the 15th of May, 1834. He had suffered several times before from similar attacks, which had been successfully treated with bleedings and other antiphlogistic remedies.

On the 17th he was admitted into the hospital, with sharp pain in the side, cough, hurried respiration, and burning fever. Four grains of cantharides, divided into twelve pills, were ordered to be taken in the course of the day.

18th. Copious perspiration during the night; pulse considerably reduced in frequency and strength; urine scanty, but voided without heat. The same dose ordered.

19th. Copious perspirations again; pulse 64 and soft; frequent desire to pass urine, and also some degree of tenesmus present; all the thoracic symptoms mitigated. The same dose repeated.

20th. Symptoms as yesterday. The urine has now become very abundant and the fever has almost entirely passed away. A scruple of camphor ordered to be added to the pills. In the course of a day or two the patient was quite convalescent.—*La Lançette Française*.

---

### Professors Oppenheim and Fricke on the Use of Iodine Injections in Hydrocele.

The advantages of injecting an iodine-tincture solution into the *tunica vaginalis* of the testicle, after the evacuation of water, are stated, by the advocates of the practice to be manifold;—the cure is much quicker, being usually effected in from three to six, instead of twelve to fourteen days as after the port-wine injection; the pain is very much less continued, the patient being often able to move about in a few hours after the operation; and the risk is very much less in the event of any of the injected fluid making its escape into the cellular tissue of the scrotum, one of the most valuable properties of iodine being its ready *absorbability*. Indeed we are told that even although several drachms or an ounce of the iodine solution be left within the *tunica vaginalis*, it will gradually be absorbed without any bad consequences.

The very numerous cases, amounting to upwards of 200, in which Mr. Martin (vide *Transactions of the Medical and Physical Society of Calcutta*) has employed the iodine solution as an injection in cases of hydrocele, afford strong testimony in favour of the practice.

Professor Oppenheim mentions that he has been induced by these successful results to give a trial to the iodine, and that he has used it with great advantage in fifteen cases.

The *first* case occurred in a countryman, sixty years of age, who had long been troubled with a hydrocele, which had been tapped several times. After drawing off about six ounces of citron-coloured fluid, Professor Oppenheim injected a mixture of one drachm of tincture of iodine and three drachms of water, and left it in the sac. As the pain was very inconsiderable, he rubbed the scrotum with his hand, so as to bring the inclosed fluid in contact with every part.

Next day, the pain was still trifling; but rather greater on the third. A few days after this, the Professor met his patient in the street apparently quite well; and on examining the scrotum a week afterwards, he found that there was no trace of any recurrence of the effusion.

The *second* case occurred in a gentleman, 40 years of age. He had been punctured three times, at intervals of from six to ten months, before he applied to Professor Oppenheim. After withdrawing the fluid, a mixture of two drachms of the iodine-tincture and six of lukewarm water was injected, and left in until considerable pain was induced. The pain continued till evening, and then subsided.

For the following two or three days, there was considerable tumefaction and uneasiness in the scrotum; but these passed away, and on the sixth day after the operation, the patient found himself so little inconvenienced that he got up, and walked a considerable distance from his house. At the end of the third week, every trace of the disease had disappeared; and up to the date of the report, fourteen months later, there had been no return of it.

The *third* case is very instructive. The patient had submitted not only to puncture of the swelling ten times, but also to the injection of port-wine into the sac, by the late Professor Delpech of Montpelier. Still the disease returned. Oppenheim now used the iodine injection—two drachms to six drachms of tepid water. The pain lasted for three or four hours and then subsided; although the testicle was a good deal swollen the next day, the patient rose from bed; and on the fifth day, he went to business without inconvenience. The cure was permanent.

The *fifth* case deserves notice on several accounts. The patient was upwards of seventy years of age; he had been troubled with the complaint on both sides for nineteen years; the operation of puncturing the sacs had been repeatedly performed; he had submitted to the introduction of a seton through the left sac, and had been confined to bed for two months in consequence of the severe pain and suffering he had endured from it;* and he had resolved never to undergo any other operation, until he accidentally heard of one of the cures effected by Professor Oppenheim. The Professor seems to have entertained some reluctance to make any attempt to effect a permanent cure in the present case, in consequence of the age of the patient, &c. But the patient being now very desirous himself, his wish was acceded to. Three drachms of iodine-tincture with an ounce and a half of tepid water were injected. After remaining for about ten minutes in the sac, no pain along the cord was induced; the Professor withdrew the canula, and left the greater part of the injection within the sac. The patient walked home after the operation. No re-action followed either on this or on any

* The use of the seton had effected a cure of the disease on this side, after very protracted suffering.

subsequent day; and a re-accumulation soon began to make its appearance. M. Oppenheim did not deem it proper to repeat the operation.

It is not necessary to go into the details of the other cases communicated by Professor Oppenheim: in all of them a cure was effected without much trouble or suffering to the patient. In none was the local reaction so strong as to require depletion. Most of the patients could walk about and attend to their business in three or four days; some in a shorter time. Usually all trace of the complaint disappeared within three or four weeks; in some within a shorter time, and in a few not for two weeks more.

The results of the practice have been so favourable that M. Oppenheim now uniformly adopts it in all cases of simple and *uncomplicated* hydrocele. It is probably from having used it in unadvisable cases, that some surgeons have reported so unfavourably of the iodine injections.

The tincture used by M. Oppenheim is prepared with 48 grains of the iodine to one ounce of alcohol. As a precipitate is speedily formed when this tincture is mixed with water, the injection should not be prepared until the moment of the operation.

With respect to the length of time that the solution should remain in the sac, this must depend altogether on the accession and severity of the pain along the cord, that may be induced. The injection is to be used tepid, as the iodine remains longer in solution, than when cold water is used.

Such is the substance of Oppenheim's report, which is highly *favorable* to the use of the iodine injections. Let us now attend to the statements of M. Fricke, who has tried the practice and has found it, he tells us, altogether *objectionable.* We shall allude briefly to his cases first, and then make a remark or two on the discrepancy between the reports of these two eminent practitioners.

The *first* case occurred in a young man, twenty-five years of age. There was a double hydrocele; and the complaint had existed for eleven years. M. *Fricke* considered that there was no disease of the testicle, nor any other complication. On the 26th Dec., the right sac was punctured, and then filled with about six drachms of a warm solution of iodine, prepared with two drachms of tincture of iodine and six ounces of distilled water. Very little local reaction followed the operation; and, by the 2nd January, the scrotum had regained its former size, and a fresh accumulation of fluid had taken place.

Four days subsequently—viz. on the 6th of January, the iodine injection was repeated. The fluid did not flow out readily, but required to be pressed out by stroking the testicle. From this circumstance M. Fricke concluded, that partial adhesion of the opposite surfaces of the tunica vaginalis had already taken place. The consequences of this second operation were most distressing. Violent fever set in, and the scrotum became first inflamed, and then partially sphacelated. A large portion of the integuments and cellular substance sloughed off; and for some time the patient was in an alarming state. Ultimately however he did well, and the hydrocele on each side was effectually removed.

The *second* case occurred in a man twenty-seven years of age. The hydrocele had followed a contusion of the testicle, and was of about six months' standing. After the fluid was discharged, a weak iodine solution, of the same strength as what was used in the former case, was injected. It proved quite ineffectual; and as the fluid re-accumulated in the sac, the operation by incision was now resorted to, and proved successful.

The *third* case was that of a youth, 19 years of age. The iodine injection speedily effected a cure.

In the *fourth* case—which occurred in a man 28 years old, and was of about six or seven months' standing—a stronger solution of iodine, (viz. one part of iodine tincture and three-parts of cold water), was used. Every thing went on well till the eighth day; and then the spermatic cord of the affected side began to swell. The swelling was followed by suppuration, which seriously retarded the recovery. Ultimately the case did quite well, and the hydrocele was got rid of.

The *fifth* case occurred in a man 42 years of age. The hydrocele was of only three weeks' standing, and did not exceed in size a hen's egg. The stronger solution of iodine—one part of the tincture and three of cold water—was used. Very trifling local reaction followed the operation. The effusion re-accumulated; and ultimately the radical operation by incision was performed.

*Sixth Case.*—A youth, 22 years old, had been affected with a double hydrocele for about four years. The stronger solution of the iodine was injected. The result was entirely successful on the right, and partially so on the left, side.

From the consideration of these cases M. Fricke is not disposed to report very favourably of the iodine injection.

He subsequently relates three cases in which he employed ice-cold water as an injection with complete success; but, on the whole, he seems to give a decided preference to the radical cure by incision, to the use of any sort of injection.—*Zeitschrift fur die Gesammte Medicin.*

*Remarks.*—It will be observed that the practice of Drs. Oppenheim and Fricke differs considerably in several points.

The impression is certainly left on our minds that the latter gentleman did not attend, in all respects, to the niceties of the operation—we mean, for example, the selection of the appropriate cases, the employment of an injection of due strength and of a proper temperature, &c. It is possible,—nay probable from the history of the case—that in the first patient the testicle was, if not positively diseased, at least highly irritable and disposed to take on a morbid action. May we not infer this from the circumstance of the hydrocele having come on at eleven years of age and having continued for 8 or 9 years? Remarks might be made on some of the other cases too, reported by M. Fricke; but this we deem unnecessary, as we prefer leaving our readers to compare facts, submitted to their attention, for themselves, and drawing their own inference from them, rather than obtruding our own personal opinion.—*Rev.*

---

### ON THE TREATMENT OF HYDROCELE WITH IODINE INJECTIONS IN THE EAST INDIES.

The following statements are from the pen of M. Dujat, who has, he informs us, recently returned from Calcutta.

Hydrocele is a disease of great frequency in the East Indies, among both the European residents, and the native inhabitants. Before the important discovery of Mr. Martin in 1832, of the efficacy of an iodine solution as an injection into the tunica vaginalis, the native inhabitants were unwilling to submit to any radical treatment, whether by the use of injections of port wine, &c. or by incision. The tediousness, as well as the severe pain, of the treatment deterred them from having anything done more than simple puncture of the swelling; and even this they often objected to, preferring to allow the hydrocele to increase to an enormous size.

It seems however that, since the year 1832, the number of patients treated in the native hospital alone at Calcutta has been very great annually. From January 1836 to Jan. 1838, 1,000 patients were admitted with hydrocele, and all of them were treated with the iodine injection. The ages of these varied from 18 to 70 years of age. The disease seems to be most frequent between the ages of 25 and 35 years. In 305 cases the hydrocele was on the right, and in 325 it was on the left, side : in the remaining 370 cases the hydrocele was double, or on both sides. The quantity of serum evacuated varied from less than ten to upwards of 100 ounces.

Of the 630 cases of single hydrocele, in rather more than one third of the whole number the quantity of serum was under ten ounces ; in *two-sevenths* it was from 10 to 20 ounces ; in nearly a *third* it was from 20 to 50 ounces ; and in eighteen cases it was from 50 to 120 ounces.

The practice recommended by Mr. Martin, and which M. Dujat saw employed in an immense number of cases, is as follows. After the serum has been discharged, the iodine solution* is injected ; and, immediately afterwards, both the canula and the syringe are removed at once, as the solution is almost always left within the sac. The surgeon then works the scrotum about in his hand, so as to bring the solution in contact with every part of the tunica vaginalis. The after-treatment consists in merely applying compresses wet with Goulard lotion to the testicle, and in administering a purgative.

The patients usually walk home immediately after the operation, and return the next day or two to the consultation.

On the second day there is, in most cases, a slight febrile reaction, and the testicle is swollen and tender.

Many patients complain for several days of a coppery taste in their mouths, and that all food is quite insipid. Some patients return to their usual occupations on the following, but most on the third or fourth, day after the operation. In a very few cases, the subsequent inflammation was rather *de trop;* but it was readily dissipated by the use of leeches, and of febrifuge medicines.

The number of failures in the 1,000 cases was extremely small ; only *once* among the cases of single hydrocele, and only six times among those where the hydrocele was double or on both sides ; and in all these last there was partial success, at least on one side. The cause of the failure was attributable either to the scrotum being affected with elephantiasis, or to the tunica vaginalis being indurated or otherwise morbid.

As some of the patients did not return after the operation, it may possibly have failed in a few of them : but this is conjectural. So satisfied are the Indian practitioners of the superiority of Mr. Martin's practice over any other, that it is now almost universally adopted.—*Gazette Medicale*, 1838.

*Remarks.*—Our readers will not fail to compare the eminently successful practice recommended by Mr. Martin, who first introduced the use of iodine injections in the treatment of hydrocele, with that of Drs. Oppenheim and Fricke, mentioned in the preceding article.

The success of Mr. Martin has indeed been very extraordinary ; and it is right to observe that his practice has been tried by M. Velpeau, who reports most favourably of it. (Vide the Foreign Periscope of this Review for April, 1838.)—*Rev.*

---

* Mr. Martin uses in almost all cases a solution of the same strength, viz. one part of the tincture (according to Magendie's formula) and three parts of water. The quantity however to be injected varies from a drachm and a half to five drachms of the mixture, according to the size of the hydrocele. The peculiarity of Mr. Martin's practice consists in using only a very small quantity of injection, and in leaving this within the sac to be absorbed by nature afterwards.

### TREATMENT OF PROLAPSUS UTERI BY PARTIAL SUTURE OF THE VULVA.

*Case* 1.—A woman, 43 years of age, was admitted into the infirmary at Hamburg in March 1837. Fifteen months before she had been cured of a prolapsus uteri by means of the operation of *Episiorraphy*—or suture of the posterior commissure of the vulva. Subsequently she had become pregnant; and as, during labour, the band of junction between the two labia appeared to oppose the delivery of the child, the accoucheur divided it across.

The consequence of this was that the prolapsus returned; and it was for the relief of this complaint that she again sought the assistance of Dr. Fricke.

The operation of *Episiorraphy* was again performed, by excising a narrow longitudinal strip of the mucous membrane of the lacerated vagina on each side, and uniting the opposed raw surfaces together by means of two or three sutures. Although the anterior and posterior ends of the wounded surfaces did not adhere, the extent of junction was quite sufficient to prevent the exit of the prolapsed uterus; and the patient left the hospital, in about six weeks after the operation, relieved of her troublesome complaint.

*Case* 2.—A woman, 41 years of age, was admitted into the infirmary on the 6th of July 1837. She reported that, six years previously, she was delivered by means of the forceps of a child, and that ever since that time she had been afflicted with a prolapsus of the uterus. The protrusion was so large, and gave so much distress, that she had been long prevented from engaging in any employment. The exposed surface of the vagina and of the cervix uteri had become dry and leathery, and was here and there excoriated and ulcerated. A constant discharge exsuded from the prolapsed parts.

After soothing and astringent means had been used for some time, in order to allay the irritation and swelling, the operation of *Episiorraphy* was performed on the 22nd of August. The commissure of the labia extended back almost to the edge of the anus; but they, the labia, were still of sufficient breadth to permit a strip of skin to be detached from them.

As it had been repeatedly observed that, in order to prevent the protrusion of the uterus and vagina, it was not absolutely necessary that the whole extent of the cleft should be made to adhere, the posterior part, or that next to the anus, was left open at present, and only the anterior part was stitched together. When the united part had cicatrised, the tendency to prolapsus was found to be completely removed, and the patient could walk about without any fear of its return. An opening between the edge of the anus and the posterior edge of the artificial commissure continued; but, as this was attended with no inconvenience, Dr. Fricke saw no propriety in doing more. Indeed he has reason to believe, from the result of this and of some other cases, that it is much better for the surgeon not to attempt the junction of the whole extent of the cleft, and that he should rather follow the practice of leaving an ununited part posteriorly—through which the menstrual secretion, &c. may find a ready exit—as adopted in the preceding case.—*Zeitschrift fur die ges: Med.*

---

### DUPUYTREN'S POMMADE AGAINST THE FALLING-OFF OF THE HAIR.

| | | |
|---|---|---|
| Take of Beef-marrow | 8 | oz. |
| Acetate of Lead | 1 | drachm. |
| Tincture of Cantharides | 1 | scruple. |
| Old Brandy | 1 | oz. |
| Oil of Cloves | 15 | drops. |

The part that is bald, or is likely soon to be so, is to be rubbed with a portion of this pommade every evening.—*Journal de Pharmacie.*

---

### Dubois' Rules for the Treatment of Uterine Hæmorrhage.

*Before Labour.* A.—If the hæmorrhage be *inconsiderable,* the following simple means may be sufficient—a horizontal posture; perfect quietude; cool air; cool acidulated drinks; evacuation of the bladder and rectum; and, if the patient be plethoric, the abstraction of a small quantity of blood from the arm.

If the hæmorrhage be *serious,* we should first have recourse to the means now mentioned, excepting perhaps that of bleeding. Should they fail, the application of cold lotions to the hypogastrium and the inside of the thighs is often serviceable; if this is insufficient, the ergot of rye* (36 grains in three doses, given at intervals of ten minutes) should be administered; and, should the hæmorrhage still continue, we must then resort to the use of the plug.†

*During Labour.*—When the hæmorrhage is *inconsiderable,* and when, at the same time, the orifice of the uterus is not dilated, and the membranes are entire, the same means recommended at A—except the blood-letting which is very rarely necessary, should be used. If the orifice is dilated, and the membranes are still entire, it will be found useful in many cases to rupture these, should the hæmorrhage continue: if the membranes are however broken, the ergot of rye is frequently useful.

When the hæmorrhage is *serious,* and the mouth of the uterus is still undilated, we are first to have recourse to the various means recommended above, including the use of cold applications to the hypogastrium, and of the ergot of rye. Should however all these means fail, we should then rupture the membranes, if this can be easily done; but if not, we must trust to the plug,‡ or proceed to extract the child either by turning or by the forceps. It is always better to trust to the efforts of Nature to expel the child, than to have recourse to manual delivery; and in most cases of hæmorrhage, except when the placenta is implanted over the orifice and neck of the uterus, this will be effected under judicious management. Even in reference to the case, where the placenta is implanted at the uterine orifice, it sometimes happens that it does not cover it completely, and that the bag of the membranes will protrude at its side, and the labour will be completed without having recourse to turning the child.

When however it is necessary to perform this operation, the fingers should be passed up between the parietes of the womb and the adhering side of the placenta, so as to detach it from its connexion, and not, as many accoucheurs recom-

---

* It is very doubtful that the hæmostatic property of this medicine depends upon its exciting the uterus to contract. It seems only to increase uterine contractions when they exist, but not to induce or bring them on, when absent.

† The operation of the plug is twofold: it first stops the hæmorrhage; and then, by its presence in the vagina, it excites the uterus to contractions, by which its orifice becomes dilated. When this takes place, we may either rupture the membranes, or allow labour to advance, according to the circumstances of the case.

‡ M. Dubois very properly points at the necessity of great attention, and of frequent examination both of the abdomen, and also, but more seldom, of the vagina, when a plug is used to arrest a serious uterine hæmorrhage. Many women have perished from internal hæmorrhage, when not a drop of blood flowed outwardly. The danger of such an occurrence is always greater when the labour-pains are weak than when they are forcible.

mend, through the substance of the latter. In a few rare instances, the placenta has actually been expelled by the natural efforts before the child, and the patient has recovered without having incurred any danger.—*La Lançette Française.*

---

### Poisoning by Arsenic successfully Treated with the Tritoxide of Iron.

A young lady, who had been long drooping from severe chagrin, attempted suicide by swallowing a quantity of arsenic. About an hour after the commission of the deed, she was seized with violent vomiting; and, as fortunately she had taken food not long before the poison was swallowed, it is probable that a considerable portion of the latter was rejected from the stomach. The excruciating pain and sense of burning in the abdomen, accompanied with cramps of the limbs, however increased; and the parents, becoming now more alarmed, called in the assistance of Dr. Deville.

From the nature of the symptoms, he at once suspected that his patient was suffering from the effects of a poison. The girl confessed the truth, and directed him to a drawer wherein the cup, from which she had drank the poison, was to be found.

After administering a quantity of milk, and having a large linseed poultice applied over the whole of the abdomen, Dr. *Deville* sent off to procure some of the *hydrated peroxide of iron*—a preparation which, within the last two years, has been strongly recommended as an antidote to the effects of arsenic. After several laboratories had been applied to in vain, a small jugful was fortunately obtained from M. *Caventon.* It was now upwards of five hours since the arsenic had been swallowed. A spoonful (nearly an ounce) of the peroxide was given every quarter of an hour, until nearly half a pound of it had been taken.

The alarming symptoms began to abate; and, as the pulse had risen and the pain of the abdomen was extremely severe, twenty-five leeches were applied to it, and the hot poultices were ordered to be persevered with.

It is unnecessary to pursue the particulars of the case further, as the patient ultimately recovered, after suffering for several days from most intense head-ache.

Dr. *Deville,* in his remarks upon the case, states that he is led to believe that very nearly a drachm of arsenic had been swallowed. True; a portion, perhaps a large one, of it had been rejected along with food by vomiting; but, as we know that even a few grains are sufficient to cause death, there is strong ground to infer that the peroxide of iron must have acted as a direct antidote, or neutralising substance.

Dr. *Bunsen* of Gottingen, who discovered this property of the peroxide, states that three or four drachms of it—recently prepared and suspended in water—to which sixteen drops of ammonia have been added, are sufficient to neutralise six or eight grains of arsenious acid: an insoluble and inert Arsenite of iron being formed.

M. *Lesneur,* while he admits the counter-acting operation of the tritoxide, says that a much larger quantity is necessary for the neutralization of the arsenic, than that stated by M. Bunsen.

But the subsequent researches of MM. *Bouley, Soubeiran,* and *Miquel,*—all able and experienced chemists—have shewn that about twelve parts of the fresh tritoxide are sufficient to neutralise one part of the arsenious acid. The sooner that it can be administered after the injection of the poison, the more prompt and effectual will be the counteraction. M. *Bouley* says that if it be taken along with the poison, it annuls the effects of the latter altogether.—*Revue Medicale.*

### Remarks on Paralysis of the Eyelids.

Although this affection is not unfrequently connected with, and is a mere outward sign and concomitant of, disease in some part of the nervous centre, it is nevertheless, very often quite a local weakness, and may be relieved by local applications,

The following four cases of *blepharoplegia* (in all, the upper eyelids were affected), are related by M. Carron du Villards, in a recent number of the *Bulletin General de Therapeutique.*

*Case* 1.—Madame Lenoir has long been subject to most troublesome attacks of *coryza.* The eyes are always more or less affected, and the lids are swollen and œdematous. After a recent attack, the right upper eyelid was found to be quite motionless; the patient had lost all power over it. The medical man in attendance had ineffectually tried various means; and the patient applied to M. *Carron.* He recommended frictions with concentrated acetic æther to be used three times a day.

By the third day the paralysis had quite disappeared.

In the *second* case, the *falling* of the upper eyelids followed an attack of erysipelas of the face, and had lasted for three weeks, when the patient applied to Mr. *Carron.* He recommended him to keep the eyelids continually wetted with a warm infusion of four drachms of the ergot of rye in boiling port wine *(quatre gros de seigle ergoté dans du vin rouge bouillant).* In two days, the eyelids had recovered their mobility.

In the *third* case, the affection of the eyelids supervened upon a partial asphyxia, induced by the power of charcoal. Fomentations, with an aqueous infusion of ergot of rye, were ordered to be kept constantly applied to the affected eyelids: in eight days the paralysis had vanished.

The *fourth* case was not quite so simple. A lady, thirty-two years of age, had been long subject to intense headaches; and latterly a complete *blepharoptosis* of the right side had come on. She had been under treatment for some time before applying to Dr. *Carron.* He ordered her to be bled in the foot; to be cupped over the nape of the neck; and to take small doses of the tartrate of antimony. The use of strychnine, both internally and externally, was now resorted to; but without advantage. A liniment, sharpened with some Croton oil, was then rubbed upon the affected eyelid and brow: this brought out a miliary eruption, and the patient began to recover speedily power over the movements of the lid.—*Bulletin General.*

---

### Cases of Glanders in the Human Subject—Discussion at the Royal Academy on.

M. Husson read the report of a case which occurred very recently at the Hôtel Dieu.

An ostler had charge of eleven horses affected with glanders: in some the disease was acute, in others it was chronic. Several of these animals were killed by order of the public authorities. The man slept in the stable along with the glandered horses.

The first symptoms of sickness in him were headache, and a profuse discharge of mucus from the nostrils. This discharge became successively sanguineous and sero-purulent. To these symptoms were soon added a violent fever and a severe pain in the right shoulder, which was considerably swollen and very tender on pressure.

Bleeding, general as well as local, was had recourse to: the blood drawn from the arm was strongly buffy. The swelling of the shoulder extended down along the whole arm. At one point was observed a gangrenous spot. Several pustules made their appearance on the body, neck, and arms; and a hard tumor was felt over the right lower ribs. The pustules became covered with eschars; delirium and coma supervened; the pulse was filiform and sometimes almost imperceptible; and death soon followed. The disease had lasted for eight days.

*Dissection.* There was observed to be a generally diffused eruption of pustules and of gangrenous spots. The pustules were large on the face especially, and on the neck and scalp. At various parts of the body there were large subcutaneous tumors, of a livid aspect on their surface. The largest of these was situated over the right shoulder, and was more than an inch in diameter. These tumors were found, on being cut into, to contain a sanguineous purulent matter. The one on the shoulder was found to communicate with another large one over the right rib by a chain of little knotty swellings. The contents of the thoracic one had made their escape into the cavity of the pleura. In many parts of the limbs, numerous abscesses were found between the muscles and the skin. Many of the lymphatic glands in the neck and elsewhere were much enlarged, and also softened.

The mucous lining of the nostrils exhibited patches of erosion and ulceration, as well as numerous pustules scattered over its surface. The *septum narium* was perforated at one point for the extent of half an inch or so, and it was covered with pustules and minute ulcerations.

The pleuræ were red; and the lungs presented the traces of suppurative lobular pneumonia.

An abscess existed in the right pleural cavity, and communicated with the swelling on the outside of the thorax.

The arteries, veins, brain, and other viscera appeared to be normal.

The pathological phenomena, which we have now enumerated as having been found in this patient, are the usual *post-mortem* appearances discovered in horses which have laboured under acute glanders.

*Case* 2nd, occurred recently in the practice of *M. Breschet.*

A robust healthy man, 34 years of age, had been employed to attend to some glandered horses, and had slept for some months in the stables where they were kept. The earliest symptom of disease in him was an attack of intense pain in the left knee; this was accompanied with swelling and redness of the part, and exhibited much of the appearance of phlegmonous erysipelas.

When taken to the Hôtel Dieu, the knee was tumefied, and covered with numerous phlyctenæ. A discharge—at first mucous, then bloody, and at last sero-purulent, and very similar to the nasal flux in glandered horses—from the nostrils came on; and, nearly about the same time, an eruption of pustules and of phlyctenæ within the nostrils, and on the fauces, neck, trunk, and arms made its appearance. The pustules were not unlike those seen in *Frambœsia*: their bases were hard, and surrounded with livid areolæ, and their apices were covered with eschars. Some of these pustules resembled small carbuncles: the cellular substance surrounding them was traversed with tracts of purulent matter. The phlyctenæ were filled with an opaque sanious matter. On examining the nasal passages, they were observed to be coated with a yellowish-coloured matter, which was readily made, by slight compression, to exsude down upon the lip. The external characters of this case may therefore be classed under four heads: 1. Pustules (like those seen in *varicella* and in *frambrœsia*) and phlyctenæ; 2. Erysipelatous swelling of the affected joint; 3. Gangrenous or carbuncular spots on the surface; and 4. Suppurations, more or less extensive, in the subdermic cellular substance.

After the eruption had made its appearance, the patient became somnolent and oppressed with stupor; there was a general *abattement* of all the corporeal energies; the urine was discharged involuntarily; the breathing was laborious and noisy from the snuffling in the nostrils; the patient was troubled with a cough; he became delirious, and died.

*Dissection.* The livid areolæ round the pustules had disappeared, and the pustules themselves were *affaissées;* their contents were a yellowish pus: the subjacent skin was excoriated. The subdermic cellular tissue was infiltrated at various points, and principally beneath the eschars, with purulent matter; and this was found also between the neighbouring tendons, and even in the substance of the muscles. The veins did not seem to be inflamed; but their contents were, in several places, found coagulated and mixed with pus. There was a sanguineous infiltration in the cellular tissue of the right arm.

The lymphatic vessels were injected, swollen, and inflamed: the glands were enlarged in the neighbourhood of the pustules. The joints were sound, with the exception of the elbow-joint, which contained a large quantity of sanguineous synovia. The mucous membrane of the nasal passages was swollen, and coated with a thick layer of *glanderous* matter. When this was carefully washed away, the membrane was found to be highly vascular and injected; here and there it exhibited small elevations formed by the concreted matter, at other points little *crypts,* such as we see on the mucous membrane of the intestines after fatal typhus fever; and mixed with these were pustules and ulcerated erosions. The submucous tissue was infiltrated with pus. The lining membrane of the maxillary and other sinuses, and that of the fauces, palate, pharynx, and even of the larynx, exhibited somewhat similar appearances. There was an abscess in the cellular tissue of the pharynx. The mucous membrane of the bronchi was highly injected, and its follicles were much enlarged. The lungs presented traces of *lobular pneumonia.* The other viscera seemed to be normal.

After the reports of these two cases had been read to the Academy, M. *Barthelemy* rose to express his dissent from the opinion of M. *Breschet,* that his case was one of genuine glanders, communicated to the patient from the horses which he had been tending. He stated that he had gone himself to the stables, where the man had been employed, and that he examined the animals very carefully; and that it was quite true that there were some glandered horses in them; but that the disease in all of them was of the chronic and not of the acute form (la morve chronique et non la morve aiguë). Now the chronic disease was, every one admitted, essentially different from the acute form, and was certainly not contagious.* It would seem however, from M. *Barthelemy's* own admission, that there had been, a fortnight before the patient's employment at the stables, a horse labouring under the acute or genuine glanders.

He urged several other objections to the idea that the disease in M. *Breschet's* case was real glanders.

M. *Rochoux* seems to have taken the same view as M. *Barthelemy,* as he expressed his disbelief in the communicability of the *morve* from the horse to the human species.

M. *Bouley* was surprised that, in the present day, any one could question the occurrence of genuine glanders in the human subject. Whether, indeed, the disease is ever of spontaneous origin, or whether in all cases it is the result of contagion from the horse, is a question which is perhaps not yet definitively settled. The latter view is the more probable; as every instance of the *glanders* hitherto observed in the human subject, has occurred in ostlers and other persons who have had to do with horses.

---

* We believe that the *chronic morve* is the *Farcy* of English veterinarians, and the *acute morve* is the genuine *Glanders.* It is unfortunate to apply the same term to two diseases, if they are different.

M. *Bouley* then canvassed the question, whether the disease is strictly contagious among horses themselves. He alluded to the results of various experiments, which had been made on this subject by a committee appointed not long ago by government, to ascertain this very point. It would seem that these experiments were considered to authorize the conclusion, that the chronic *morve* is not contagious; and even that the acute form is not always, but only occasionally, so. In very many cases it was found, when healthy horses were brought into contact or at least contiguity with glandered horses, there was no communication of the disease. M. *Bouley* seems to lean to the idea, that the disease, such as appeared in M. *Breschet's* case, and which, of late years, has been by many decided to be glanders communicated from diseased horses, may possibly be of spontaneous origin in the human constitution.

M. *Rayer* replied to the preceding speakers. He adduced various arguments to prove that the glanders in the human subject* is a disease *sui generis*, and distinct from any other in the nosological catalogue; and that it is strictly analogous in its essential phenomena—the leading symptoms during life, and the pathological appearances discoverable on dissection—to the same disease in horses. In both, there is a morbid state of the nasal passages; in both there is an eruption of pustules and gangrenous spots on the skin; in both, there is frequently a lobular pneumonia; and in both, there is the rheumatic affection, and also the appearance of purulent tumors in different parts of the body.

The lungs, the nasal passages, and the skin, are the organs which are most generally and most seriously diseased in glanders. The immediate cause of death may usually be traced to the purulent alterations of the lungs. The proposal, therefore, to perform tracheotomy in such cases is founded on an erroneous presumption.

M. *Rayer* dwelt very forcibly on the acknowledged fact that all the recorded cases of glanders in the human subject have occurred in persons who have had to deal with horses. He then proceeded to discuss the assertion, made by M. *Bouley* and others, that the chronic form of the disease is not contagious. His experience and researches are directly opposed to the truth of this opinion. The one form is frequently observed to be convertible into the other: if this be the case, are we not bound to admit that they are strictly of the same nature?

The conclusions, which M. *Bouley* had drawn from the experiments recently made by the French Committee, are, in M. *Rayer's* opinion, quite erroneous. In the last place, the learned speaker alluded to several experiments, narrated on most trust-worthy authority, which shew that, on the one hand, the inoculation of the matter—(it is not stated whether it be the nasal discharge, or the contents of the pustules on the skin)—from the human subject is capable of producing genuine glanders in the horse; and, on the other hand, the too frequent occurrence of veterinary surgeons, and others, accidentally catching the disease from glandered horses, sufficiently proves the converse of the position.

MM. *Velpeau* and *Blandin* followed M. *Rayer*, supporting the same view of the question, and expressing their decided belief that the disease in the two cases reported above was genuine glanders, and was caught by infection from diseased horses.—*Memoires de l'Academie de Medicine.*

---

* Dr. Elliotson, we believe, has proposed to designate the disease *Equinia*, to tally with *Vaccinia*. It seems to be a very good appellation.

A Consultation of MM. Rayer, Orfila, and Caffe, on a Case of Milky Urine: Chemical Examination of the Urine and Blood: Practical Remarks.

M. *Costa*, 22 years of age, a native of Brazil, stated that, four years ago, he first observed that his urine had occasionally a milky appearance: his general health at the time was very good, and he experienced no uneasiness in any part of the urinary apparatus. A year afterwards he began to suffer attacks of severe pain in the region of the kidneys and in that of the bladder: the pain was frequently excessive before and during the evacuation of his water, causing him to bend himself forwards and draw up his limbs to his stomach. The urine at this time was extremely thick, so that it flowed out with difficulty, and had often a quantity of blood mixed with it.

These attacks usually lasted for about twelve or fourteen days, and were treated with copious leeching of the hypogastrium and perineum, warm baths, and the use of nitred drinks. The cessation of the urinary distress was slow and gradual, and was not complete for upwards of three months; the state of the urine varied every now and then; being at one time milky, at another time natural, and at a third more or less bloody.

From this period, the urine often remained quite clear and normal for two or three months at a time, and then re-assumed a milky appearance without any appreciable cause. The exercise of riding seemed generally to favour the re-establishment of a healthy state of the secretion.

While at *Rio Janeiro* he had taken a quantity of terebinthinate preparations, and was kept on a light unstimulating course of diet.

In July 1836, he came to Paris with the view of consulting some of the leading physicians there for his malady; which, although it had not visibly affected his health, was nearly stationary and made him very uneasy as to the consequences. His urine had been quite natural during the whole of the voyage to France.

On reaching Paris, he consulted M. *Caffe*, who recommended *at first* a decoction of willow-twigs to be alternated with the waters of Vichy, Barege-baths, warm clothing to the surface, and avoidance of exposure to cold; and *subsequently* a decoction of horse-radish, and pills of sulphate of iron and sub-carbonate of potash. These means seemed to have no effect on the complaint.

Towards the end of September, M. Costa visited Belgium and England, continuing the use of the chalybeates during the whole of the time. The travelling certainly benefited him; but the urine again assumed a milky aspect shortly before he returned to Paris.

MM. *Orfila* and *Rayer* now joined M. *Caffe* in consultation upon this curious case; and the following memoranda of the symptoms were marked down.

The urine was very seldom more abundant than the quantity of liquid drank. The milky colour varied exceedingly at different times, both in intensity and durability: at one time the urine was nearly as white as milk; then it was only somewhat troubled, and at length it assumed a perfectly healthy and limpid aspect. These changes rapidly succeeded each other without any appreciable cause. Even when milky, the urine had always its characteristic smell and taste. Since the use of the chalybeate medicines, it had been much less frequently bloody than it was before; and no pain in voiding it had been experienced of late. The general health of the patient seemed to be perfectly good; his appetite was regular and vigorous, and his sleep was always sound. The urine was generally clear for some time after sexual intercourse.

M. *Guibourt*, professor of pharmaceutical chemistry at the *Ecole de Medicine*, was requested to analyse the urine at different times; and the following is the substance of his report to the three physicians in attendance.

"The urine of M. *Costa* is sometimes white like milk; at other times it is of a blood-red colour; and at other times again it is quite clear and of a healthy appearance.

When it is red, if left to repose, a dark red deposit is found to subside to the bottom of the vessel,* the supernatant liquid being then always more or less decidedly white and turbid.

The milky urine contains sometimes so much fatty matter, that the surface becomes, after a short repose, covered with a thick layer, like cream. If sulphuric æther be added to the milky urine, it quickly becomes nearly quite clear, and the æther acquires a yellow colour. By evaporating this æther, a quantity of fatty matter may be readily obtained.

The milky urine, which has been made transparent by the addition of sulphuric æther, forms an abundant coagulum of albumen, on being boiled. Nitric acid also coagulates it; but acetic acid does not. This proves that there is no *caseine* in the urine; and hence that, strictly speaking, it should not be called *milky*.

The milky urine, after it was freed from all the fatty matter by means of æther, and also from its albumen by boiling, was found, on the addition of nitric acid, to deposit beautiful crystals of nitrate of urea. We therefore conclude that the urine of M. Costa differs from healthy natural urine, only in containing a quantity of fatty matter and of albumen, to which may also be added the occasional admixture of the colouring matter of the blood, superadded to the usual ingredients of the secretion."

The results of M. *Guibourt's* analyses, if compared with those by *Dr. Prout* of what he calls *chylous* urine, shew very clearly that the present case is to be considered as one of this kind. That the term *chylous* is more appropriate than that of *milky*, appears from the circumstances that no *caseine* can be detected in the secretion, and that, if we add a small portion of genuine chyle to healthy urine, we obtain a fluid which will be found to contain albumen, fatty matter, and sometimes also a certain proportion of the colouring matter of the blood along with the usual ingredients of urine.

The attention of MM. *Rayer, Orfila,* and *Caffe* was now directed to ascertain the state of the blood in their patient.

Four ounces were drawn from the arm, and *M. Guibourt* was again requested to lend his assistance.

The blood, after some hours' repose, was found to be a gelatinous trembling mass, without any appearance of white buff (*couenne blanche*) on the surface. After twenty-four hours, when shaken about in a glass vessel, it became completely fluid—a circumstance which seemed to indicate an utter absence of fibrine. This liquid blood was mixed with two parts of alcohol, for the purpose of coagulating it: the coagulum was then pressed and dried. It weighed seven and a half drachms, or nearly one-fourth of the weight of the blood. It was pulverulent, and of a pale reddish-white colour; whereas the coagulum of healthy blood, after being treated in the same manner, is a hard vitreous-looking substance of a very deep red-brown colour.

It appears therefore that the blood of M. *Costa* contains much less colouring matter than healthy blood does. It is to be remarked however, that this difference might be partly owing to the large quantity of albumen present in the former; for certainly the quantity of dried coagulum—seven and a half drachms from four ounces of blood—exceeds that usually met with in the analysis of healthy blood; and as this coagulum was found to contain very little *fibrine,* we must suppose that the solid matter was chiefly *albumen.*

By subsequent experiments made with æther on the dried coagulum of M. *Costa's* blood, and on the same quantity of dried coagulum of healthy blood, it was found that about four grains of a fatty, solid, opaque substance was ob-

* This deposit, although it much resembled a coagulum of blood, is not strictly so. It consists of the colouring matter only, without any of the fibrine. This is readily proved by adding some æther, which completely dissolves it and leaves no trace of the deposit.

tained from the former, and only two grains of a coloured residue, partly fatty and partly saline, from the latter.

From all these researches we may fairly conclude that the blood of M. *Costa* differed from healthy blood in containing less fibrine, and a greater quantity of albuminous and fatty matter; and therefore that it more nearly approached to the composition of chyle than the latter.

The practical application of these conclusions is, that the alteration of the urinary secretion may be considered as dependent upon an abnormal state of the circulating fluid—a state which seems to depend upon an imperfect transformation or assimilation of the chyle with the blood. The case of M. *Costa* was therefore considered by M. *Rayer* and his colleagues as one of primary vitiated sanguification or *hematosis.* The treatment which these gentlemen recommended was as follows:—

1. The patient to take fasting every morning, during several months, six steel pills.*

2. To take an ounce of cinchona wine every day, an hour before dinner.

3. To use a cool bath, to which two ounces of sulphate of potash have been added, for half an hour at a time, thrice a week.

4. Every evening before going to bed, to take twenty-four grains of subcarbonate of iron in a wafer or in stewed fruit, (dans du pain à chanter ou dans de la compote.)

5. The food to consist, in a great part, of beef or mutton, either roasted or grilled.

6. The ordinary beverage to be of some generous wine *coupé* with a chalybeate water.

7. Sea-bathing during the fine weather.

8. The occasional attacks of strangury being attributable altogether to a mechanical obstruction of the urinary passages from coagula, recourse must be had to diluents, and, if necessary, to the use of the catheter to relieve this symptom.

Before concluding these remarks on this rare affection, we may briefly state

1. That in the cases of chylous urine mentioned by Dr. Prout, the patients, although some of them had been subject to the irregularity for several years, did not exhibit any marked disturbance of the general health.

2. That the cases of *hæmaturia,* which are of frequent occurrence in certain tropical climates as in the Isle of France, and are not unfrequently followed by a milky or chylous state of the urine, are not usually regarded as attended with much danger.

From the report of a discussion, which recently took place in the Academy of *Rio Janeiro,* the metropolis of *Brazil,* it appears that this abnormal state of the urinary secretion is not of unfrequent occurrence there,† especially among females; and that the gravity or danger of the affection is by no means commensurate with its tediousness and resistance to therapeutic measures.

3. Cases of milky or chylous urine are rare in Europe. In those, of which we have any report, the general health of the patients does not seem to have been much disordered. In some, however, the affection seems to have been followed by *diabetes.*

---

* The formula for these was—

Take of Subcarbonate of iron.... ℨj.

P. cinchonæ rubræ .... ℈j.

P. canellæ ............ gr. xij.

M. ft. misce in pil. xxiv. div.

† It seems that the late Ex-Emperor of Brazil, *Don Pedro,* had consulted M. *Rayer,* in consequence of a milky state of his urine. It is not stated whether the health of the royal invalid suffered from the disorder.

The preceding details are extracted from a report which is signed with the names of MM. *Caffe, Orfila,* and *Rayer.*—*Le Presse Medicale.*

---

### UTILITY OF IRON (ESPECIALLY THE IODURET) IN SOME SYPHILITIC AFFECTIONS.

M. *Ricord* of the Hôpital des Veneriens at Paris has, for some time past, used pretty extensively the *proto-ioduret of iron* in cases of secondary syphilis, when the system of the patient is feeble, and especially if at the same time it is scrofulous. He reports very favourably of its beneficial effect under such circumstances.

In numerous instances where the ordinary methods of treatment had been ineffectually tried, the constitution seemed to rally quickly under the use of this preparation of steel, and the local disease at the same time began to assume a healthy action. Repeatedly have we seen, says M. *Ricord,* old extensive ulcers of the throat, which had long resisted all the usual topical applications and internal medicines, rapidly change their aspect under the influence of the *proto-ioduret,* and, within a very short time, heal and cicatrize. In many patients affected with caries of the bones of the face, cranium, and extremities, the process of separation of the diseased from the healthy parts has been surprisingly quickened, and the general health has at the same time been materially improved by its use.

Another set of symptoms, which has been much benefited by the use of the proto-ioduret, deserves to be especially noticed—we allude to old tedious discharges from the vagina and urethra, occurring in persons of enfeebled lymphatic constitutions. The dose, which M. *Ricord* recommends, is six grains during twenty-four hours at first: this is to be gradually increased to half a drachm or two scruples. Besides the internal administration of the ioduret, its external use, as an injection, has been extensively adopted by M. *Ricord* in cases of obstinate gleet. The strength of the solution is usually half a drachm to eight ouuces of water: in a few cases the quantity of the ioduret has been raised to two drachms.

As illustrative of the beneficial influence of steel upon certain forms of venereal ulceration, we may here appropriately mention that M. *Cullerier* has frequently used the carbonate of iron with happy results in cases of unhealthy ulcerated buboes.

The following case furnishes an appropriate example.

A middle-aged man was admitted into the Hôpital du Midi with numerous chancres on the penis, and a very large bubo in the right groin. A blister was applied over this swelling, and next day the denuded surface was covered with lint wetted in a strong solution of sulphate of copper (ʒj. to ℥j. of water:) an eschar was formed, and when this separated, a quantity of pus was discharged. The swelling however remained almost as large as ever; and friction with mercurial ointment was therefore resorted to. The gums becoming painful, the ointment of the hydriodate of potash was substituted. Subsequently the kali causticum was applied, to cause another opening in the tumor. The ulcerated bubo began to assume a most unpromising aspect; its edges being everted, and its base black and oozing blood. Leeches were applied several times on its inner surface, but without producing any benefit. (*Is not this rather a strange practice*). —*Rev.*

The patient, after three months' suffering, was greatly reduced in flesh and strength; the ulcers were fungoid and livid; the gums were spongy and bled on the slightest pressure; and the general health seemed to indicate a scorbutic cachexia. M. *Cullerier* now recommended the internal use of the carbonate of iron, and, by continuing it for a month, the man was discharged quite well.—*La Lançette Française.*

